# A Systematic Review of Methods, Study Quality, and Results of Economic Evaluation for Childhood and Adolescent Obesity Intervention

**DOI:** 10.3390/ijerph16030485

**Published:** 2019-02-08

**Authors:** Mandana Zanganeh, Peymane Adab, Bai Li, Emma Frew

**Affiliations:** Institute of Applied Health Research, College of Medical and Dental Sciences, University of Birmingham, Birmingham B15 2TT, UK; MXZ666@student.bham.ac.uk (M.Z.); P.ADAB@bham.ac.uk (P.A.); B.Li.3@bham.ac.uk (B.L.)

**Keywords:** cost effectiveness, methods, children, adolescents, obesity, prevention, treatment

## Abstract

Many suggested policy interventions for childhood and adolescent obesity have costs and effects that fall outside the health care sector. These cross-sectorial costs and consequences have implications for how economic evaluation is applied and although previous systematic reviews have provided a summary of cost-effectiveness, very few have conducted a review of methods applied. We undertook this comprehensive review of economic evaluations, appraising the methods used, assessing the quality of the economic evaluations, and summarising cost-effectiveness. Nine electronic databases were searched for full-economic evaluation studies published between January 2001 and April 2017 with no language or country restrictions. 39 economic evaluation studies were reviewed and quality assessed. Almost all the studies were from Western countries and methods were found to vary by country, setting and type of intervention. The majority, particularly “behavioural and policy” preventive interventions, were cost-effective, even cost-saving. Only four interventions were not cost effective. This systematic review suggests that economic evaluation of obesity interventions is an expanding area of research. However, methodological heterogeneity makes evidence synthesis challenging. Whilst upstream interventions show promise, an expanded and consistent approach to evaluate cost-effectiveness is needed to capture health and non-health costs and consequences.

## 1. Introduction

Childhood obesity is a major global public health problem with associated health, social, and emotional consequences, as well as long term direct and indirect costs [1,2,3,4]. Effective obesity prevention and treatment in children and adolescents is therefore a priority as it is far more cost-effective to prevent the onset of obesity in childhood compared to a lifetime of obesity-associated costs. However, despite an increasing number of intervention studies, there are relatively few published economic evaluations [5,6,7].

In many countries, the scarcity of public resources requires decision makers to seek information on cost-effectiveness as well as clinical effectiveness in the knowledge that the use of resources in one way prevents their use in others [8]. Economic evaluation is a means to aid decisions about public resource allocation [9,10] and as obesity prevention and treatment often involves lifestyle interventions that have costs and consequences that fall outside the health care sector, a societal perspective for evaluation is usually recommended [10]. This means that all relevant resource use/costs and consequences are measured, outlining how these fit within a given sector, such as health, education, or the wider community [11]. However, when incorporating costs and outcomes that span across multiple sectors, it is not always clear how much society is willing to pay for a ‘non-health’ effect caused by an intervention funded from a ‘health care budget’. Also, the valuation of resources for which no market exists, such as informal care, or patient time costs, requires specific methods [8].

Seven recent reviews [12,13,14,15,16,17,18] have summarised the cost-effectiveness of obesity prevention and/or treatment interventions in young people however none were designed to offer a rigorous review of methods applied for economic evaluation. Five reviews had language restrictions [12,13,14,15,17] and four excluded studies that were conducted in developing countries [12,13,15,17], limiting global interpretation. Only two reviews appraised methods for handling inter-sectoral costs [13,17]. Just three of the reviews used established criteria, e.g., Drummond checklist [19] to assess the quality of the primary studies [13,14,17]. The search strategy was inadequate (e.g., search terms not fully reported) in three reviews [12,15,18], and in the remaining four there were omissions of relevant databases, which means that relevant studies could have been missed [13,14,16,17]. Furthermore, the most recent review, which only focused on interventions in pre-school children, included studies reported up to November 2015 and, at least three new economic evaluation studies of childhood obesity interventions have been published since then [20,21,22].

This paper reports on a systematic review of published economic evaluations of obesity prevention and/or treatment interventions in children and adolescents (0–19 years) with the primary objective of appraising the methods used and assessing the quality of the economic evaluations using the Drummond checklist [19]. A secondary objective was to undertake a narrative synthesis of the evidence of the cost-effectiveness.

## 2. Materials and Methods

The systematic review follows the reporting guidelines of Preferred Reporting Items for Systematic Reviews and Meta-Analyses (PRISMA) [23] and a completed PRISMA checklist is presented in Section A (see Supplementary Materials). The protocol is registered with the international prospective register of systematic reviews (PROSPERO) database ref (CRD42017062236) and has previously been published [24].

### 2.1. Literature Search

The following electronic health economics/biomedical databases were searched: MEDLINE (Ovid); EMBASE (Ovid); Web of Science; CINAHL Plus; EconLit; PsycINFO; Cochrane Database of Systematic Reviews (CDSR); Database of Abstracts of Reviews of Effects (DARE); the National Health Service Economic Evaluation Database (NHS EED); Health Technology Assessment (HTA) and Cost-Effectiveness Analysis (CEA) Registry. The following sources were also used to identify potential additional studies: Google Scholar; relevant National Institute for Health and Care Excellence (NICE) guidelines; the reference lists of eligible studies and review articles; and Grey literature such as OpenSIGLE, National Obesity Observatory, NHS Evidence, National Technical Information Service, Healthcare Management Information Consortium (HMIC), and RePEC (Economic Working papers) database. The search was conducted in May 2017 and studies were sought between January 2001 and April 2017. The year 2001 was chosen since the first study evaluating the cost-effectiveness of a childhood obesity treatment intervention was published then followed two years later by the first economic evaluation of a childhood obesity prevention intervention [16]. Search strategies included Medical Subject Headings (MeSH) terms and text words of key papers that were identified beforehand. The search terms and text words were adapted for use within other bibliographic databases. The full search strategy is presented in Section B (see Supplementary Materials).

### 2.2. Inclusion and Exclusion Criteria

Economic evaluations were included or excluded based on the following criteria:

- Types of study: Primary full economic evaluations were included (studies in which both the costs and outcomes of the alternatives are examined and in which a comparison of two or more interventions or case alternatives are undertaken) including trial-based and model-based (using trial data) evaluations. Partial economic evaluations; qualitative studies; conference abstracts; and study protocols were excluded.
- Participants/population: Children and adolescents aged 0–19 years at the start of the intervention and/or their parents/guardians were included. Family based interventions were also included when the target participants were the children. Economic evaluations undertaken within any country context were included. Interventions to tackle obesity due to a secondary cause (e.g., Prader–Willi syndrome) were excluded.
- Intervention(s), exposure(s): All behavioural (focused on individual behaviour change techniques), environmental (focused on modifying the local environment), or policy (focused on population-wide legislative or fiscal action) interventions for the treatment or prevention of overweight/obesity in children and/or adolescents were included. Pharmacological or surgical interventions were excluded.
- Comparator(s)/control: Only studies with a clearly defined comparator were included with no restrictions on the types of comparator(s).
- Outcome(s): No restrictions on outcomes measures. Potentially relevant outcomes were: disability-adjusted life years (DALYs); quality-adjusted life years (QALYs); effectiveness outcomes such as kilogramme weight loss; % body fat; body mass index (BMI) z-score; waist circumference; overweight and obesity cases avoided; additional minute of moderate to vigorous physical activity (MVPA); increase in overall physical activity level and metabolic equivalent (MET) hour gained.

Other criteria: There were no restrictions based on language.

### 2.3. Study Selection Procedure

The review followed a two-stage method. First, the main researcher and an independent researcher individually screened titles and abstracts of identified publications against the selection criteria. If in doubt, the full text version was requested. Second, full-text papers were reviewed by both researchers and a final decision made with respect to the inclusion/exclusion criteria. There was 85% agreement between the two reviewers. Any disagreements between the reviewers over the eligibility of specific studies were resolved by discussion between all authors. To aid study selection and analysis of non-English language articles, translation either in part or in whole was undertaken by academic colleagues with the appropriate language skills. The literature search results were managed using EndNote X7 (Thomson Reuters, Philadelphia, PA, USA).

### 2.4. Data Extraction

The study characteristics and findings were recorded using a standardised, pre-piloted data extraction form (see Tables S1 (i)–S1 (iv) and Tables S2-4 (i)–S2-4 (iv) in Supplementary Materials). This process was checked for completeness and accuracy by an independent researcher. Any discrepancies between the reviewers over the data extraction process was identified and resolved by discussion or by consensus with all authors.

### 2.5. Quality Assessment of Included Studies

The quality of the economic evaluations were judged against standard criteria (Drummond checklist) [19], see Table S5 (see Supplementary Materials). Quality assessment of the included studies was independently checked for completeness and accuracy by an independent researcher and any discrepancies were resolved by discussion with all authors.

## 3. Results

**Figure 1:**
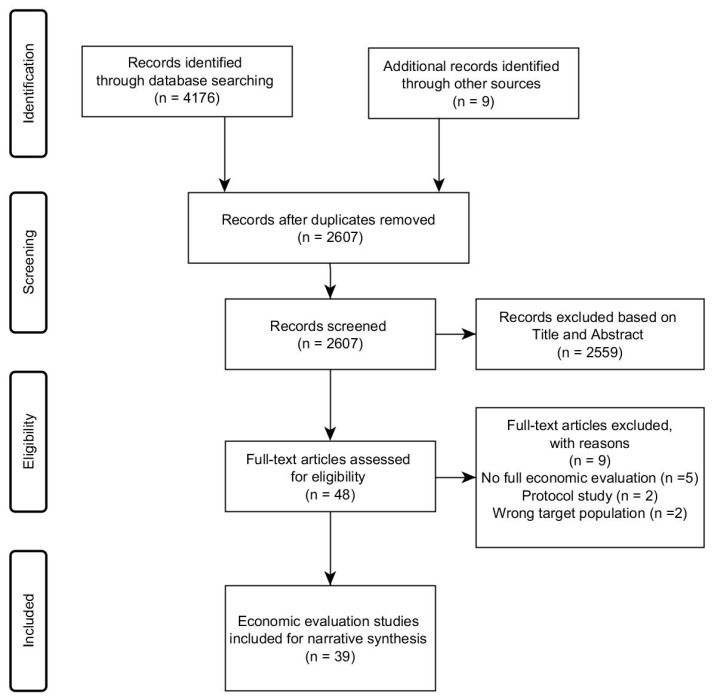
PRISMA (Preferred Reporting Items for Systematic Reviews and Meta-Analyses) flow diagram.

From the 4185 references initially identified, 39 economic evaluations were included—see Figure 1. The most common reasons for exclusion were the lack of (full) economic evaluations, being a protocol study, or including an ineligible target population.

### 3.1. Details about Study Context

Full details about study context are presented in Tables S1 (i)–S1 (iv) (see Supplementary Materials), and summarised in Table 1.

#### 3.1.1. Intervention and Comparator

**Figure 2:**
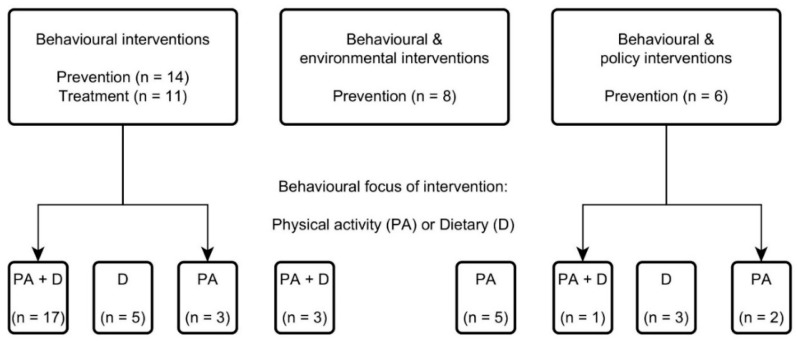
Summary of the interventions.

Approximately half of the economic evaluations (23/39) were model-based [12,22,25,26,27,28,29,30,31,32,33,34,35,36,37,38,39,40] compared to trial-based evaluations. A range of interventions were identified, all containing individual behaviour change elements (Figure 2). A large proportion (25/39) (including all treatment interventions) were focused exclusively on behaviour change techniques, the rest combined individual behaviour change elements with either an environmental component (modifying the local environment e.g., active school transport) [21,29,30,31,32,33,41,42] or a policy component (population-wide legislative or fiscal interventions such as banning unhealthy food advertising or a physical education policy) [25,27,28,35,38,43]. Approximately half of the interventions (21/39; 12 prevention and 9 treatment) targeted a combination of physical activity and dietary behaviours [12,20,26,32,36,37,38,39,40,41,42,44,45,46,47,48,49,50], the rest focused on either physical activity [21,25,29,30,31,33,43,51,52,53] or dietary habits only [12,22,27,28,35,54].

The intensity of the interventions differed considerably. For prevention interventions, this ranged from one session per three months [44] to approximately two sessions per month [51]; and for treatment interventions, ranged from 1 [20] to 12 sessions per week [46]. The duration of the interventions also differed, ranging from eight months [53] to four years [52] for prevention studies; and from three months [20] to one year [46] for treatment studies. Overall therefore, the treatment interventions were generally more intensive but delivered over a shorter time period compared to prevention interventions. The comparison or control group was not always clearly specified but was assumed to be ‘usual care’ in most of the studies (33/39) and often the studies did not justify their rationale for choosing the comparator.

#### 3.1.2. Country and Setting

The evaluations were spread across a wide range of countries and study settings. The vast majority (38/39) originated from high-income countries, mainly Australasia (Australia (n = 15) [12,21,28,29,30,31,32,40,44,50] and New Zealand (n = 2) [34,41]), with 13 from North America (USA (n = 12) [22,25,26,27,35,36,37,38,42,43,46,49], Canada (n = 1) [47]) and 8 from Europe (UK (n = 4) [20,33,39,48], Germany (n = 2) [51,52], Finland (n = 1) [54], Spain (n = 1) [53]). Only one study was from a developing country context—China [45]. In terms of study setting, the majority of prevention interventions (21/28) were school-based [21,22,26,29,30,31,32,34,36,37,41,42,45,49,51,52,53] and for treatment interventions, most (9/11) took place in clinical settings [12,20,39,40,46,47,48,50,54].

### 3.2. Review of Economic Evaluation Methods

A detailed account of the economic evaluation methods are presented in Tables S2-4 (i)–S2-4 (iv) (see Supplementary Materials).

#### 3.2.1. Type of Economic Evaluation and Measures of Effectiveness

The majority of studies that conducted a cost-effectiveness analysis (CEA) used raw or standardised BMI as a measure of clinical outcome (26/39) (18 prevention and 8 treatment), whilst other studies used one or more measures from: cases of overweight/obesity prevented; unit increase in MET minutes; reduction in body fat or waist circumference. Approximately half of the studies that undertook a CEA also conducted a cost-utility analysis (CUA) [20,27,28,29,30,31,32,33,35,40] with QALYs as the primary outcome measure. The review found that the vast majority of trial-based economic evaluations (15/16) did not use QALYs/DALYs whereas most model-based evaluations (n = 20) tended to report QALYs/DALYs as the primary economic outcome. When QALYs were used, the age of the participants was between 6 and 11 years in the trial-based economic evaluation [20], and between 2 and 19 years in the model-based economic evaluations.

A pattern with preferred type of economic evaluation by country context was apparent. Within Australasia (13/17) a CUA or a combination of CUA and CEA [12,28,29,30,31,32,34,40] was most popular, whereas the majority of studies from North America (7/13) [25,38,42,43,46,47,49], and the only study from China [45] conducted a CEA only. Across Europe, only UK-based studies used CUA [20,33]. In terms of study setting, a CEA was most common in clinical settings (7/9), whereas within school settings a mixed approach was applied with around half conducting a CEA (12/22). There was no clear pattern found in terms of approach taken to evaluate prevention or treatment interventions.

#### 3.2.2. Evaluation Perspective Taken

Most (35/39) studies clearly reported the study perspective. The majority (n = 29) were from a societal perspective. Interestingly, none of the UK studies [20,33,39,48], compared to most of those conducted within Australia and the USA, applied a societal perspective. Two studies reported using a health care perspective, but from the data reported it was clear that wider societal costs were included within a secondary analysis [20,50].

For all interventions that included either a policy or environmental component (12/14), the perspective was societal, whereas for interventions focused exclusively on individual behaviour change a combination of societal (17/25) and healthcare (6/25) perspectives was undertaken. A societal perspective was also adopted by the vast majority of interventions implemented in school settings (19/22).

#### 3.2.3. Time Horizon Considered and Type of Modelling Approach Taken

The time durations for the trial-based economic evaluations were predicted by the period of the trial. Of interest this ranged from 8 months [53] to 6 years [52] in the prevention studies, and from 10 months [49] to 15 months [50] for the treatment interventions. For the model-based evaluations, the time horizon was more at the analysts discretion and within this review ranged from at least 10 years (n = 5) [25,27,35,37,38] to a lifetime (15/23) [12,22,28,29,30,31,32,34,39,40]. The time horizon was also found to be much shorter within clinical settings (6/9) [20,46,47,48,50,54] compared to the other study settings such as schools for example. The majority of the studies did not justify their choice of time horizon.

With respect to modelling, the vast majority of model-based studies (18/23) applied Markov modelling [12,25,27,28,29,30,31,32,34,35,38,39,40] compared to decision analytic modelling [22,26,33,36,37]. The majority of the model-based studies did not justify their model choice and the description of model details was suboptimal in most of them.

#### 3.2.4. Choice of Discount Rate

For the majority of the trial-based studies (10/16) (4 prevention and 6 treatment), discounting was not appropriate as the time horizons considered were relatively short (less than one year) [20,42,45,46,47,48,49,51,53,54]. For all the trial-based studies of more than one year, all reported using a discount rate in accordance with the relevant country guidelines apart from one prevention trial from New Zealand [41], which used a 5% discount rate per year for costs, rather than the 3.5% discount rate per year for both costs and outcomes recommended [55]. Most model-based studies (22/23) applied a discount rate for both costs and outcomes (3% per year for Australia (n = 12) [12,28,29,30,31,32,40], the USA (n = 8) [22,25,26,27,35,36,37,38] and the UK (n = 1) [39], and 3.5% per year for New Zealand (n = 1) [34]. Interestingly, the rates used for studies from Australia and the UK were not in accordance with their respective country guidelines (which is 5% per year for Australia according to PBAC and 3.5% per year for the UK according to NICE) [56,57]. However, different state governments in Australia recommend different rates and the discount rate used in the included Australian and UK studies was consistent with the US panel recommendations [58]. Most of the studies did not justify their choice of discount rate.

#### 3.2.5. Methods for Collecting and Estimating Resource Use/Costs

Half of the trial-based evaluations (8/16) (four prevention and four treatment) reported their methods for collecting resource use [20,21,44,46,48,50,51,52], while only 10 out of 23 model-based evaluations (nine prevention and one treatment) did so [22,25,27,29,30,31,32,35,38,40].

As expected, the choice of inclusion of a particular type of cost varied considerably according to the study purpose, perspective, setting and the nature of the intervention being evaluated. Costs tended to be categorised into programme delivery, direct medical (e.g., healthcare visits), direct non-medical (e.g., travel time/cost for participants) and indirect (e.g., productivity losses because of parents’ absence from work). In line with recommendations for CEA [59], the development/set up costs were not considered in the vast majority of studies, apart from one trial-based prevention study from the USA [43].

Of the nine studies (five prevention and four treatment) that included indirect costs incurred by parents [20,29,30,32,40,42,46,50,52], these were mainly from Australia (n = 5) and most of them were for preventive ‘behavioural’ interventions within a school-based setting (5/9). Also, direct non-medical costs were reported by four prevention studies from Australia [29,32,40,50] and one treatment study from the USA [46]. Most of these types of costs (3/5) were for ‘behavioural’ interventions implemented within a clinical setting.

#### 3.2.6. Sensitivity Analysis Undertaken

The majority of the trial-based studies (10/16) conducted a deterministic sensitivity analysis to assess the robustness of the results [20,21,41,42,44,50,51,52,53,54]. Most of the model-based studies (22/23) apart from the study by Pringle et al. (2010) from the UK [33], conducted at least one type of sensitivity analysis with the majority (n = 20) applying both deterministic and probabilistic sensitivity analysis in line with recommendations. Half of these studies, however, did not justify the choice of covariates for the sensitivity analysis.

### 3.3. Narrative Synthesis of Cost-Effectiveness Evidence

The most common method for presenting cost-effectiveness evidence was the incremental cost-effectiveness ratio (ICER) (30/39). The vast majority of the studies (33/37), excluding the CCA ones, reported results that were cost-effective. Some of these (13 of the model-based prevention/treatment studies including 5 by Carter et al. (2009)), [12,27,28,33,35,36,37,38,39] illustrated cost saving results. For instance, Long et al. (2015) concluded that a sugar-sweetened beverage excise tax would increase benefits in terms of DALYs averted and result in healthcare cost savings in the USA [27]. Almost half of these 13 studies that illustrated cost-savings were from Australia, followed by 5 from the USA and 2 from the UK. None of the trial-based evaluations reported cost saving results, probably due to shorter time horizons. Whilst the findings are not directly comparable between studies due to the heterogeneous nature of the methods used, all of the studies which evaluated interventions targeting only dietary habits (8/8) and the majority of the studies targeting both physical activity and dietary habits (19/21) indicated cost-effective or cost saving results. However, the studies which focused on only physical activity indicated a proportionally smaller number of cost-effective or cost saving results (7/10). Furthermore, the evidence suggests that the majority of behavioural interventions supported by a policy intervention (4/6) were cost-saving [27,28,35,38].

A small number of studies (n = 4) [20,29,30,31] reported interventions to not be cost-effective. The UK trial-based treatment study [20], which targeted a combination of physical activity and dietary habits with the aim to reduce weight gain in children with obesity remained not cost-effective using a CEA/CUA approach regardless of the choice of perspective. Also, the three model-based studies that targeted only physical activity were not cost-effective, for example, the “Walking School Bus” programme which had a high cost of delivery coupled with low participation rates [29].

### 3.4. Quality Assessment of the Included Studies

The quality of reporting the economic evaluations was assessed using the Drummond checklist. Full details of the quality assessment are presented in Tables S6 (i)–S6 (iv) (see Supplementary Materials). None of the included studies fulfilled all of the quality criteria however only a small number of the studies were categorised as poor. One challenge regarding the quality assessment was that quality was judged based on the published data only and there might be a difference in what has been reported and what has actually been done. So a bad scoring study might just be due to lack of transparency rather than lack of quality.

Certain criteria were simply not applicable to each respective study (e.g., items 12–15, due to different perspectives chosen), while others were not reported. The three criteria which were least well addressed were the rationale for the comparator, the justification for the choice of discount rate, and the model choice. Whilst the time horizon for each study was generally well specified, most studies omitted to provide reasons for choice. Additionally, approximately half of the studies did not justify the choice of economic evaluation nor offered justification for what was explored within a sensitivity analysis.

## 4. Discussion

To the best of our knowledge, this is the first study to conduct a review of the methods for economic evaluation and to determine how these methods vary by setting, country, and intervention design.

The review identified some emerging patterns. We found that among the published economic evaluations, there was no consistent measure of outcomes. Around half of the studies reported clinical (e.g., BMI), rather than health-related outcome measures commonly used within economic evaluation (QALYs/DALYs). This suggests that the measurement of QALYs/DALYs within obesity trials is not firmly established. This heterogeneity of outcome measures will hinder comparability of cost-effectiveness.

No evaluation applied a cost–benefit analysis (CBA) approach. Consideration of broader outcomes going beyond the health sector allows for inclusion of costs and effects from multiple sectors and is particularly relevant for obesity intervention. This is an emerging area of development within economic evaluation and efforts are being made to adapt methodologies to promote the use of CBA [60]. These approaches have been recommended by the UK Treasury guidance to evaluate (usually non-health) public sector projects [61].

Model-based evaluations offer the opportunity to improve the generalisability of results as they combine data from a variety of sources. However the findings from five of the model-based evaluations identified within this review were based on small samples [22,26,33,36,37] and only one of these offered data based on a lifetime horizon. Furthermore, all of the model-based evaluations were for interventions that targeted individual health behaviours and were therefore highly dependent on cultural, infrastructural and other system-related aspects. So the generalisability of results to other contexts, particularly from developed to developing country settings, would be questionable [62]. The majority of the papers did not make explicit mention of procedures for checking their models and no study assessed the sensitivity of their results to the choice of model-type. Despite associated assumptions with modelling studies, the studies evaluated are important as model-based health economic evaluations are today widely accepted as policy-making tools that can inform resource allocation decisions. Almost half of the model-based studies chose a lifetime perspective and the vast majority of them applied Markov modelling.

Most trial-based and model-based evaluations in this review applied recommended discount rates in accordance with the relevant country guidelines. Methods for collecting resource use and the type of cost included were found to vary across the studies. In particular, the indirect costs of overweight and obesity (e.g., productivity losses) were not generally collected alongside the trials. It is considered good practice to report results both with and without indirect costs. Including indirect costs (e.g., costs incurred by families) has the potential to alter the treatment recommendations.

The narrative synthesis of the economic evidence and the quality assessment of the included studies are useful for informing health economists/modellers and the direction for future research in this area. In terms of judging cost-effectiveness of interventions, context-specific assessment is problematic as there are different thresholds for cost-effectiveness in different countries. For example, in the UK, NICE recommends a threshold willingness to pay of £20,000–30,000 per QALY [63], by contrast in Australia the recommendation is AU$50,000 per QALY [29] and in many countries there are no clearly defined thresholds at all. Whilst most interventions in this review appear cost-effective using standard rules of cost-effectiveness, there is substantial variation by intervention design.

### 4.1. Comparison with Previous Systematic Reviews

Our finding that most interventions were cost-effective or even cost-saving, is similar to those reported by two other reviews [14,18], with some overlap between included studies. Other reviews have focused on particular age groups (e.g., pre-schoolers [13]), specific interventions (e.g., only physical activity [17]), or particular outcomes (e.g., anthropometric measurements [13]). Two additional reviews from Australia [12] and the US [15] used the assessing cost-effectiveness (ACE) obesity approach to summarise and compare the cost-effectiveness of a range of interventions. However, none of the previous studies reviewed the methods of the economic evaluations in the way we have outlined.

### 4.2. Strengths and Limitations of this Review

One of the important strengths of this review is the comprehensive search strategy applied encompassing a broad range of electronic bibliographic databases of published studies and the grey literature (six additional studies were identified). Furthermore, the results were not limited to only those published in English (two non-English publications identified) and there were no country restrictions (there was one publication from china as a developing country), resulting in a more complete review than those published previously. Also, the formal quality assessment of the economic evaluations undertaken adds strength to the conclusions. The vast majority of the studies were found to be of very good reporting quality.

The review had some limitations. As we focused on full economic evaluations, some important data contained within partial evaluations may have been missed. Further limitations relate to the shortcomings of the included studies and underlying evidence base. There was heterogeneity in both the methods used and with the type of intervention being evaluated, which made synthesising the evidence base challenging. Not all included studies used the same definition of obesity, which may impact on the results. Most of the included studies reported an economic evaluation for an intervention that had previously been reported as clinically effective. It is possible that any trial which had ineffective results did not conduct an economic evaluation or, if they did, failed to get it published, introducing potential publication bias.

## 5. Conclusions

This systematic review suggests that current economic evaluations are mainly set in developed countries and the majority focus on the prevention of obesity in children, compared to treatment. Our findings show that the majority of published economic evaluations are for interventions with an individual behaviour change component. The majority, particularly ‘behavioural and policy’ preventive interventions, were cost-effective, even cost-saving. However the review found heterogeneity with respect to methods applied. So, to improve the evidence base further and to enhance comparability across interventions, we recommend a consistent and expanded form of economic evaluation which captures both health and non-health costs and consequences beyond health-gain.

## Figures and Tables

**Table 1:** Summary of general characteristics of the studies.

Study Characteristics		Number of Studies Identified (%)
**Year of publication**		
2001–2009		17 (44)
2010–2017		22 (56)
**Study approach**		
Trial-based	Prevention	9 (23)
	Treatment	7 (18)
Model-based	Prevention	19 (49)
	Treatment	4 (10)
**Comparator selected**		
Usual care		33 (85)
Another intervention		6 (15)
**Country**		
**High-income**		
Australia		15 (38.5)
New Zealand		2 (5)
The USA		12 (31.5)
Canada		1 (2.5)
The UK		4 (10)
Germany		2 (5)
Finland		1 (2.5)
Spain		1 (2.5)
**Low and middle-income**		
China		1 (2.5)
**Setting**		
Prevention		
School		21 (54)
US/Australian state		5 (13)
Community		1 (2.5)
Home		1 (2.5)
Treatment		
Clinical		9 (23)
School		1 (2.5)
Community		1 (2.5)

## Supplementary Materials

Additional supporting information may be found online in the supplementary material file for this article at http://www.mdpi.com/1660-4601/16/3/485/s1. This file includes: Section A: Completed PRISMA checklist; Section B: Search strategy; Tables S1 (i)–S1 (iv): Data extraction (Details about study context); Tables S2-4 (i)–4 (iv): Data extraction (Detailed account of the economic evaluation methods); Table S5: Drummond checklist for critically appraising relevant studies; and Tables S6 (i)–S6 (iv): Quality assessment of the included studies.
