# Supplementary material for: A Systematic Review of Methods, Study Quality, and Results of Economic Evaluation for Childhood and Adolescent Obesity Intervention
Source: Int J Environ Res Public Health. 2019 Feb 8;16(3):485. doi: 10.3390/ijerph16030485 (PMC6388206; doi:10.3390/ijerph16030485)
Supplement: Supplementary file 1 [file ijerph-16-00485-s001.pdf]

A systematic review of methods, study quality, and results of economic evaluation for childhood and adolescent obesity intervention

Mandana Zanganeh, Peymane Adab, Bai Li and Emma Frew

Supplementary materials

Section A: Completed PRISMA checklist

Section B: Search strategy

Tables S1 (i) – S1 (iv): Data extraction (Details about study context)

Tables S2-4 (i) – S2-4 (iv): Data extraction (Detailed account of the economic evaluation methods)

Table S5: Drummond checklist for critically appraising relevant studies

Tables S6 (i) – S6 (iv): Quality assessment of the included studies

Section A: Completed PRISMA checklist

Section/topic	#	Checklist item	Reported on page #
**TITLE**			
Title	1	Identify the report as a systematic review, meta-analysis, or both.	1
**ABSTRACT**			
Structured summary	2	Provide a structured summary including, as applicable: background; objectives; data sources; study eligibility criteria, participants, and interventions; study appraisal and synthesis methods; results; limitations; conclusions and implications of key findings; systematic review registration number.	1-2
**INTRODUCTION**			
Rationale	3	Describe the rationale for the review in the context of what is already known.	3-4
Objectives	4	Provide an explicit statement of questions being addressed with reference to participants, interventions, comparisons, outcomes, and study design (PICOS).	4
**METHODS**			
Protocol and registration	5	Indicate if a review protocol exists, if and where it can be accessed (e.g., Web address), and, if available, provide registration information including registration number.	4
Eligibility criteria	6	Specify study characteristics (e.g., PICOS, length of follow-up) and report characteristics (e.g., years considered, language, publication status) used as criteria for eligibility, giving rationale.	5-6
Information sources	7	Describe all information sources (e.g., databases with dates of coverage, contact with study authors to identify additional studies) in the search and date last searched.	4-5
Search	8	Present full electronic search strategy for at least one database, including any limits used, such that it could be repeated.	Supplementary material (Section B)
Study selection	9	State the process for selecting studies (i.e., screening, eligibility, included in systematic review, and, if applicable, included in the meta-analysis).	6
Data collection process	10	Describe method of data extraction from reports (e.g., piloted forms, independently, in duplicate) and any processes for obtaining and confirming data from investigators.	6

Data items	11	List and define all variables for which data were sought (e.g., PICOS, funding sources) and any assumptions and simplifications made.	4-6
Risk of bias in individual studies	12	Describe methods used for assessing risk of bias of individual studies (including specification of whether this was done at the study or outcome level), and how this information is to be used in any data synthesis.	N/A
Summary measures	13	State the principal summary measures (e.g., risk ratio, difference in means).	N/A
Synthesis of results	14	Describe the methods of handling data and combining results of studies, if done, including measures of consistency (e.g., I^2) for each meta-analysis.	6-7
Risk of bias across studies	15	Specify any assessment of risk of bias that may affect the cumulative evidence (e.g., publication bias, selective reporting within studies).	N/A
Additional analyses	16	Describe methods of additional analyses (e.g., sensitivity or subgroup analyses, meta-regression), if done, indicating which were pre-specified.	N/A
**RESULTS**			
Study selection	17	Give numbers of studies screened, assessed for eligibility, and included in the review, with reasons for exclusions at each stage, ideally with a flow diagram.	7
Study characteristics	18	For each study, present characteristics for which data were extracted (e.g., study size, PICOS, follow-up period) and provide the citations.	7-10+ Supplementary material **(Tables S1 (i) – S1 (iv))**
Risk of bias within studies	19	Present data on risk of bias of each study and, if available, any outcome level assessment (see item 12).	N/A
Results of individual studies	20	For all outcomes considered (benefits or harms), present, for each study: (a) simple summary data for each intervention group (b) effect estimates and confidence intervals, ideally with a forest plot.	7-10+ Supplementary material (**Tables S1 (i) – S1 (iv)**)
Synthesis of results	21	Present results of each meta-analysis done, including confidence intervals and measures of consistency.	10-14+ Supplementary material (**Tables S2-4 (i) – S2-4 (iv)**)

Risk of bias across studies	22	Present results of any assessment of risk of bias across studies (see Item 15).	N/A
Additional analysis	23	Give results of additional analyses, if done (e.g., sensitivity or subgroup analyses, meta-regression [see Item 16]).	N/A
**DISCUSSION**			
Summary of evidence	24	Summarize the main findings including the strength of evidence for each main outcome; consider their relevance to key groups (e.g., healthcare providers, users, and policy makers).	14-16
Limitations	25	Discuss limitations at study and outcome level (e.g., risk of bias), and at review-level (e.g., incomplete retrieval of identified research, reporting bias).	16
Conclusions	26	Provide a general interpretation of the results in the context of other evidence, and implications for future research.	16
**FUNDING**			
Funding	27	Describe sources of funding for the systematic review and other support (e.g., supply of data); role of funders for the systematic review.	17

From: Liberati A, Altman DG, Tetzlaff J, et al. The PRISMA statement for reporting systematic reviews and meta-analyses of studies that evaluate health care interventions: explanation and elaboration. PLoS Medicine. 2009;6(7):e1000100

For more information, visit: **www.prisma-statement.org**

Section B: Search strategy

MEDLINE (Ovid)

1. exp Obesity/
2. Obese.mp.
3. exp Overweight/
4. (BMI or body mass index).af.
5. Weight gain/
6. (Overweight or over weight or obesity or adipose).af.
7. exp Child/
8. exp Infant/
9. (Child* or adolescen* or infant*).af.
10. Schoolchild*.mp.
11. exp Adolescent/
12. (Boys or girls or youth or youths).af.
13. (Teenage* or young person).af.
14. (Nutrition adj2 intervent*).af.
15. (Obesity adj2 prevent* or treat*).af.
16. Counsel?ing.mp.
17. exp support groups/
18. exp Health Behaviour.mp.
19. exp Life Style/
20. exp Delivery of Health Care/
21. exp Social Support/
22. exp Family Practice/
23. exp Parent-Child Relations/
24. Food Habits .mp.
25. exp Diet therapy/
26. exp Food Preferences/
27. exp Exercise therapy/
28. Physical activit*.mp.
29. Economic Evaluat*.mp.
30. Cost*.ti.
31. Cost?Benefit*.mp.
32. Cost?Utilit*.mp.
33. Cost?Effective*.mp.
34. exp "costs and cost analysis"/
35. 1 or 2 or 3 or 4 or 5 or 6
36. 7 or 8 or 9 or 10 or 11 or 12 or 13
37. 14 or 15 or 16 or 17 or 18 or 19 or 20 or 21 or 22 or 23 or 24 or 25 or 26 or 27 or 28
38. 29 or 30 or 31 or 32 or 33 or 34
39. 35 and 36 and 37 and 38
40. Limit 39 to (yr=”2001-Current”)

EMBASE (Ovid)

1. exp Obesity/
2. Obese.mp.
3. exp Overweight/
4. (BMI or body mass index).af.
5. Weight gain/
6. (Overweight or over weight or obesity or adipose).af.

7. exp Child/
8. exp Infant/
9. (Child* or adolescen* or infant*).af.
10. Schoolchild*.mp.
11. exp Adolescent/
12. (Boys or girls or youth or youths).af.
13. (Teenage* or young person).af.
14. (Nutrition adj2 intervent*).af.
15. (Obesity adj2 prevent* or treat*).af.
16. Counsel?ing.mp.
17. exp support groups/
18. exp Health Behaviour.mp.
19. exp Life Style/
20. exp Delivery of Health Care/
21. exp Social Support/
22. exp Family Practice/
23. exp Parent-Child Relations/
24. Food Habits .mp.
25. exp Diet therapy/
26. exp Food Preferences/
27. exp Exercise therapy/
28. Physical activit*.mp.
29. Economic Evaluat*.mp.
30. Cost*.ti.
31. Cost?Benefit*.mp.
32. Cost?Utilit*.mp.
33. Cost?Effective*.mp.
34. exp "costs and cost analysis"/
35. 1 or 2 or 3 or 4 or 5 or 6
36. 7 or 8 or 9 or 10 or 11 or 12 or 13
37. 14 or 15 or 16 or 17 or 18 or 19 or 20 or 21 or 22 or 23 or 24 or 25 or 26 or 27 or 28
38. 29 or 30 or 31 or 32 or 33 or 34
39. 35 and 36 and 37 and 38
40. Limit 39 to (yr=”2001-Current”)

PsycINFO

1. exp Obesity/
2. Obese.mp.
3. exp Overweight/
4. (BMI or body mass index).af.
5. Weight gain/
6. (Overweight or over weight or obesity or adipose).af.
7. exp Child/
8. exp Infant/
9. (Child* or adolescen* or infant*).af.
10. Schoolchild*.mp.
11. exp Adolescent/
12. (Boys or girls or youth or youths).af.
13. (Teenage* or young person).af.
14. (Nutrition adj2 intervent*).af.
15. (Obesity adj2 prevent* or treat*).af.
16. Counsel?ing.mp.

17. exp support groups/
18. exp Health Behaviour.mp.
19. exp Life Style/
20. exp Delivery of Health Care/
21. exp Social Support/
22. exp Family Practice/
23. exp Parent-Child Relations/
24. Food Habits .mp.
25. exp Diet therapy/
26. exp Food Preferences/
27. exp Exercise therapy/
28. Physical activit*.mp.
29. Economic Evaluat*.mp.
30. Cost*.ti.
31. Cost?Benefit*.mp.
32. Cost?Utilit*.mp.
33. Cost?Effective*.mp.
34. exp "costs and cost analysis"/
35. 1 or 2 or 3 or 4 or 5 or 6
36. 7 or 8 or 9 or 10 or 11 or 12 or 13
37. 14 or 15 or 16 or 17 or 18 or 19 or 20 or 21 or 22 or 23 or 24 or 25 or 26 or 27 or 28
38. 29 or 30 or 31 or 32 or 33 or 34
39. 35 and 36 and 37 and 38
40. Limit 39 to (yr=”2001-Current”)

Web of Science

1. TS= (Obesity OR obese OR overweight)
2. TS= (Child OR infant OR schoolchild* OR adolescent)
3. TS= (Interven* OR prevent* OR therapeutics OR counseling OR “primary health care” OR “preventive health services” OR “health behaviour” OR “life style” OR “health knowledge, practice, attitudes” OR “delivery of health care” OR “social support” OR “family practice” OR “parent-child relations” OR “food habits” OR “food preferences” OR exercise OR sports)
4. TS= (“Economic evaluat*” OR costs* OR “cost?benefit*” OR “cost?utilit*” OR “cost?effective*”)
5. #1 AND #2 AND #3 AND #4 Timespan 2001-2017

CINAHL Plus

S1. (MH “Obesity+”)
S2. “obese”
S3. “overweight”
S4. (MH "Child+")
S5. (MH "Infant+")
S6. "schoolchild*"
S7. "adolescent"
S8. "Interven*"
S9. "prevent*"
S10. (MH "Therapeutics+")
S11. "counseling"
S12. (MH "Primary Health Care")
S13. "preventive health services"
S14. (MH "Health Behavior")

S15. (MH "Life Style+")
S16. "health knowledge, practice, attitudes"
S17. "delivery of health care"
S18. "social support"
S19. (MH "Family Practice")
S20. (MH "Parent-Child Relations")
S21. (MH "Food Habits")
S22. (MH "Food Preferences")
S23. (MH "Exercise+")
S24. (MH "Sports+")
S25. "Economic evaluat*"
S26. "costs*"
S27. "cost?benefit*"
S28. “cost?utilit*”
S29. “cost?effective*”
S30. S1 OR S2 OR S3
S31. S4 OR S5 OR S6 OR S7
S32. S8 OR S9 OR S10 OR S11 OR S12 OR S13 OR S14 OR S15 OR S16 OR S17 OR S18 OR S19 OR S20 OR S21 OR S22 OR S23 OR S24
S33. S25 OR S26 OR S27 OR S28 OR S29
S34. S30 AND S31 AND S32 AND S33
S35. Limit S34 to Publication Year: 2001-2017

EconLit

S1. (MH “Obesity+”)
S2. “obese”
S3. “overweight”
S4. (MH "Child+")
S5. (MH "Infant+")
S6. "schoolchild*"
S7. "adolescent"
S8. "Interven*"
S9. "prevent*"
S10. (MH "Therapeutics+")
S11. "counseling"
S12. (MH "Primary Health Care")
S13. "preventive health services"
S14. (MH "Health Behavior")
S15. (MH "Life Style+")
S16. "health knowledge, practice, attitudes"
S17. "delivery of health care"
S18. "social support"
S19. (MH "Family Practice")
S20. (MH "Parent-Child Relations")
S21. (MH "Food Habits")
S22. (MH "Food Preferences")
S23. (MH "Exercise+")
S24. (MH "Sports+")
S25. "Economic evaluat*"
S26. "costs*"
S27. "cost?benefit*"
S28. “cost?utilit*”

S29. “cost?effective*”
S30. S1 OR S2 OR S3
S31. S4 OR S5 OR S6 OR S7
S32. S8 OR S9 OR S10 OR S11 OR S12 OR S13 OR S14 OR S15 OR S16 OR S17 OR S18 OR S19 OR S20 OR S21 OR S22 OR S23 OR S24
S33. S25 OR S26 OR S27 OR S28 OR S29
S34. S30 AND S31 AND S32 AND S33
S35. Limit S34 to Publication Year: 2001-2017

CRD (DARE, NHS EED, HTA)

1. MeSH DESCRIPTOR Obesity EXPLODE ALL TREES
2. (Obese) OR (Overweight): any field
3. MeSH DESCRIPTOR Child EXPLODE ALL TREES
4. MeSH DESCRIPTOR Infant EXPLODE ALL TREES
5. (Schoolchild*): any field
6. MeSH DESCRIPTOR Adolescent EXPLODE ALL TREES
7. (Interven*) OR (prevent*): any field
8. MeSH DESCRIPTOR Therapeutics EXPLODE ALL TREES
9. MeSH DESCRIPTOR Counseling EXPLODE ALL TREES
10. MeSH DESCRIPTOR Primary Health Care EXPLODE ALL TREES
11. MeSH DESCRIPTOR Preventive Health Services EXPLODE ALL TREES
12. MeSH DESCRIPTOR Health Behavior EXPLODE ALL TREES
13. MeSH DESCRIPTOR Life Style EXPLODE ALL TREES
14. (Health knowledge, practice, attitudes): any field
15. MeSH DESCRIPTOR Delivery of Health Care EXPLODE ALL TREES
16. MeSH DESCRIPTOR Social Support EXPLODE ALL TREES
17. MeSH DESCRIPTOR Family Practice EXPLODE ALL TREES
18. MeSH DESCRIPTOR Parent-Child Relations EXPLODE ALL TREES
19. (Food Habits): any field
20. MeSH DESCRIPTOR Food Preferences EXPLODE ALL TREES
21. MeSH DESCRIPTOR Exercise EXPLODE ALL TREES
22. MeSH DESCRIPTOR Sports EXPLODE ALL TREES
23. MeSH DESCRIPTOR Costs and Cost Analysis EXPLODE ALL TREES
24. MeSH DESCRIPTOR Economics EXPLODE ALL TREES
25. (Cost) OR (Economic): any field
26. (#1 or #2) and (#3 or #4 or #5 or #6) and (#7 or #8 or #9 or #10 or #11 or #12 or #13 or #14 or #15 or #16 or #17 or #18 or #19 or #20 or #21 or #22) and (#23 or #24 or #25) From 2001-2017

CENTRAL and CDSR

1. "MeSH descriptor: [Obesity] explode all trees
2. Obese or overweight: ti, ab.kw (Word variations have been searched)
3. "MeSH descriptor: [Child] explode all trees
4. "MeSH descriptor: [Infant] explode all trees
5. Schoolchild*: ti, ab.kw (Word variations have been searched)
6. "MeSH descriptor: [Adolescent] explode all trees
7. Interven* or prevent*: ti, ab.kw (Word variations have been searched)
8. "MeSH descriptor: [Therapeutics] explode all trees
9. "MeSH descriptor: [Counseling] explode all trees
10. "MeSH descriptor: [Primary Health Care] explode all trees
11. "MeSH descriptor: [Preventive Health Services] explode all trees

12. "MeSH descriptor: [Health Behavior] explode all trees
13. "MeSH descriptor: [Life Style] explode all trees
14. Health knowledge, practice, attitudes: ti, ab.kw (Word variations have been searched)
15. "MeSH descriptor: [Delivery of Health Care] explode all trees
16. "MeSH descriptor: [Social Support] explode all trees
17. "MeSH descriptor: [Family Practice] explode all trees
18. "MeSH descriptor: [Parent-Child Relations] explode all trees
19. "MeSH descriptor: [Food Habits] explode all trees
20. "MeSH descriptor: [Food Preferences] explode all trees
21. "MeSH descriptor: [Exercise] explode all trees
22. "MeSH descriptor: [Sports] explode all trees
23. "MeSH descriptor: [Costs and Cost Analysis] explode all trees
24. "MeSH descriptor: [Economics] explode all trees
25. Cost or economic: ti, ab.kw (Word variations have been searched)
26. (#1 or #2) and (#3 or #4 or #5 or #6) and (#7 or #8 or #9 or #10 or #11 or #12 or #13 or #14 or #15 or #16 or #17 or #18 or #19 or #20 or #21 or #22) and (#23 or #24 or #25) From 2001-2017, in other reviews or economic evaluations

Tables S1 (i) – S1 (iv): Data extraction (Details about study context)

Table S1 (i) Details about study context (trial-based prevention studies) (alphabetically sorted)

Authors	Year	Country	Study design	Setting	Target population/ age group	N (analytical sample)	Parents/ guardians included	Intervention overview /target	Intervention aim /mode of delivery	Comparator
Hayes et al.	2014	Australia	RCT	Home	Up to age 2 years, boys/girls from a mixed-weight group	324 parents with infants	Yes: Not for indirect and direct non-medical costs	8 one-to-one consultations with education and advice on feeding, nutrition and physical activity	Prevention /nurse	Usual care, plus home safety information sent by mail
Kesztyus et al.	2011	Germany	RCT	School	7-8 years, boys/girls from a mixed-weight group	945 children	Yes: parents involved but not costed	28 units, health education, physical activity breaks	Prevention /teacher	Usual care
Krauth et al.	2013	Germany	Cohort	School	6-10 years, boys/girls from a mixed-weight group	660 children	Yes	3 additional lessons per week regarding physical activity	Prevention /teacher	Usual care
Martinez et al.	2011	Spain	RCT	School	9-10 years, boys/girls from a mixed-weight group	1,409 children	Yes: parents involved but not costed	3 sessions, school-based physical activity program	Prevention /teacher	Usual care
McAuley et al.	2010	New Zealand	RCT	School-community	5–12 years, boys/girls from a mixed-weight group	279 children	Yes: parents involved but not costed	A pilot program for nutrition and physical activity	Prevention /activity coordinator	Usual care

Table S1 (i) Details about study context (trial-based prevention studies) (alphabetically sorted) continued

Meng et al.	2013	China	RCT	School	6-13 years, boys/girls from a mixed-weight group	8, 301 children	Yes: parents involved but not costed	6 times, nutrition education for children, parents and teachers, physical activity intervention and comprehensive intervention.	Prevention /teacher	Usual care
Peterson et al.	2008	USA	Cross-sectional	State	12–18 years, boys/girls from a mixed-weight group	3,782 adolescents	No	The get up and do something media campaign (Television and/or billboards) for physical activity	Prevention (policy) /media	Usual care
Sutherland et al.	2016	Australia	RCT	School-community	13-16 years, boys/girls from a mixed-weight group	1,150 adolescents	Yes: Not for indirect and direct non-medical costs	Seven physical activity promotion strategies and six additional strategies	Prevention /trained teacher	Usual care
Wang et al.	2008	USA	RCT	School (after hours)	6-10 years, boys/girls from a mixed-weight group	182 children	Yes	After school environment program: physical activity, healthy snacks	Prevention /coordinator	Usual care

Notes: RCT = randomised controlled trial

Table S1 (ii) Details about study context (trial-based treatment studies) (alphabetically sorted)

Authors	Year	Country	Study design	Setting	Target population/ age group	N (analytical sample)	Parents/ guardians included	Intervention overview /target	Intervention aim /mode of delivery	Comparator
Epstein et al.	2014	USA	RCT	Primary care	8-12 years, boys/girls with obesity/ overweight	50 children with parents	Yes	Family-based behavioural treatment, 15 sessions (12 weekly, 2 biweekly and 1 monthly): diet, physical activity and behaviour change for both treatment groups	Treatment /staff	Separate group treatment (parent and child)
Goldfield et al.	2001	Canada	RCT	Primary care	8-12 years, boys/girls with obesity	24 children with parents	Yes: Not for indirect and direct non-medical costs	Group treatment, 13 sessions (8 weekly, 4 bi-weekly, and 1 monthly): diet, physical activity and behaviour change for both treatment groups	Treatment /counselling degree	Mixed family-based behavioural treatment
Hollinghurst et al.	2013	UK	RCT	Primary care/ home/ hospital	5-16 years and 9-17 years, boys/girls with obesity	143 children and adolescents	Yes: Parents involved but not costed	Every 3 months, nurse-led, input from dietitian and exercise specialist and an intensive intervention Mandometer	Treatment /doctor, nurse, exercise specialist, dietitian	Hospital, Consultant-led care with discretionary input from dietitian and exercise specialist

Table S1 (ii) Details about study context (trial-based treatment studies) (alphabetically sorted) continued

Janicke et al.	2009	USA	RCT	Community	8-14 years, boys/girls with obesity/ overweight	76 children	Yes: Not for indirect and direct non-medical costs	Parent only behavioural intervention, group sessions: weekly: 8, bi-weekly: 4/ diet and physical activity for both treatment groups	Treatment /post-doctoral psychologist and graduate students in clinical psychology	Family-based behavioural intervention,
Kalavainen et al.	2009	Finland	RCT	Primary care	7–9 years, boys/girls with obesity	70 children with parents	Yes: Not for indirect and direct non-medical costs	Routine counselling treatment, 2 appointments for children: diet	Treatment /nurses, nutritionists	Group treatment, 15 separate sessions for parents and children: diet
Robertson et al.	2017	UK	RCT	NHS primary care	6-11 years, boys/girls with obesity/ overweight	128 children with 137 parents/ carers	Yes	1 per week, parenting skills, social and emotional development/ physical activity and diet	Treatment /intervention team	Usual care
Wake et al.	2008	Australia	RCT	Primary care	5–9 years, boys/girls with obesity/ overweight	163 children with parents	Yes	Training of GP (3 times 2.5 h), 4 consultations over a 12-week period/physical activity, diet	Treatment /GP	Usual care

Notes: GP = general practitioner; RCT = randomised controlled trial

Table S1 (iii) Details about study context (model-based prevention studies) (alphabetically sorted)

Authors	Year	Country	Study design	Setting	Target population/ age group	N (analytical sample)	Parents/ guardians included	Intervention overview /target	Intervention aim /mode of delivery	Comparator
Barrett et al.	2015	USA	Cohort	State's school	6–11 years, boys/girls from a mixed-weight group	17.6 million children	No	Active physical education policy (Active PE)	Prevention (policy) /teachers	Usual care
Brown et al.	2007	USA	Cohort	School	8-11 years, Boys/girls from a mixed-weight group	423 children	No	Physical education, school food service modification, family- and home-based program	Prevention /teacher, trainer	Usual care
Carter et al.	2009	Australia	RCT	School	7–11 years, boys/girls from a mixed-weight group	595, 000 children over 5 years (119,000 each year)	Yes: Parents involved but not costed	Education programme to reduce sugar sweetened drink consumption	Prevention /trained project staff	Usual care
Carter et al.	2009	Australia	Cohort	School	6 years, boys/girls from a mixed-weight group	114, 630 children	Yes: Parents involved but not costed	Education to improve nutrition and physical activity, with an active physical education	Prevention /teacher	Usual care

Table S1 (iii) Details about study context (model-based prevention studies) (alphabetically sorted) continued

Carter et al.	2009	Australia	RCT	School	8–10 years, boys/girls from a mixed-weight group	268, 600 children	Yes: parents involved but not costed	Education programme to reduce television viewing of snacks	Prevention /teacher	Usual care
Carter et al.	2009	Australia	Cohort	School	6 years, boys/girls from a mixed-weight group	114, 630 children	Yes: parents involved but not costed	Education to improve nutrition and physical activity, without an active physical education	Prevention /teacher	Usual care
Graziose et al.	2016	USA	RCT	School	10-11 years, boys/girls from a mixed-weight group	769 children	No	24 lessons, obesity prevention nutrition education	Prevention /trained teacher	Usual care
Long et al.	2015	USA	Cohort	State	2-19 years, boys/girls from a mixed-weight group	74 million children	No	Sugar-sweetened beverage excise tax/diet	Prevention (policy) /government, industry	Usual care
Magnus et al.	2009	Australia	RCT	State	5-14 years, boys/girls from a mixed-weight group	2.4 million children	Yes: parents involved but not costed	Removing TV advertising of energy-dense nutrition-poor (EDNP) food and beverages,	Prevention (policy) /media	Usual care

Table S1 (iii) Details about study context (model-based prevention studies) (alphabetically sorted) continued

Moodie et al.	2009	Australia	Cohort	School-community	5-7 years, boys/girls from a mixed-weight group	7, 840 children	Yes	Walking school bus program, encouraging physical activity	Prevention /volunteer conductors	Usual care
Moodie et al.	2010	Australia	Cohort	School (after hours)	5-11 years, boys/girls from a mixed-weight group	99, 000 children	Yes	Active after-school communities, physical activity	Prevention /organizations	Usual care
Moodie et al.	2011	Australia	Cohort	School-community	10–11 years, boys/girls from a mixed-weight group	267, 700 children	Yes: parents involved but not costed	Travel smart school, promotion of physical activity	Prevention /teachers	Usual care
Moodie et al.	2013	Australia	Quasi-experimental,	School-community	4-12 years, boys/girls from a mixed-weight group	2, 184 children	Yes	The be active eat well program, diet and physical activity	Prevention /community service	Usual care
Pringle et al.	2010	UK	_	Community	10-17 years, boys/girls from a mixed-weight group	343 children and adolescents	Yes: Not for indirect and direct non-medical costs	Free swimming activities, campaigns, exercise classes, motivational interviews	Prevention /trainer	Other interventions
Rush et al.	2014	New Zealand	RCT	School	6-8 years and 9-11 years, boys/girls from a mixed-weight group	2, 474 younger and 2, 330 older children	Yes: parents involved but not costed	Project Energize: Multicomponent physical activity and nutrition	Prevention /organizations	Usual care

Table S1 (iii) Details about study context (model-based prevention studies) (alphabetically sorted) continued

Sonneville et al.	2015	USA	Cohort	State	2-19 years, boys/girls from a mixed-weight group	74 million Children and adolescents	No	Elimination of the tax subsidy of TV advertising costs for nutritionally poor foods and beverages advertised	Prevention (policy) /industry	Usual care
Wang et al.	2003	USA	RCT	School	10-14 years, girls from a mixed-weight group	620 children	No	Lessons, sport materials, wellness, teacher training, targeting diet and physical activity, reduction of TV viewing time	Prevention/ teacher, trainer	Usual care
Wang et al.	2011	USA	RCT	School	10-14 years, girls from a mixed-weight group	480 children	No	Lessons, sport materials, wellness, teacher training, targeting diet and physical activity	Prevention/ teacher, trainer	Usual care
Wright et al.	2015	USA	Cohort	State	2.5-5 years, boys/girls from a mixed-weight group	3.7 million children	No	Early care and education policy change, physical activity, diet and reduction of TV viewing time	Prevention (policy) /Child care trainers	Usual care

Notes: RCT = randomised controlled trial

Table S1 (iv) Details about study context (model-based treatment studies) (alphabetically sorted)

**Authors**	**Year**	**Country**	**Study design**	**Setting**	**Target population/ age group**	**N (analytical sample)**	**Parents/ guardians included**	**Intervention overview /target**	**Intervention aim /mode of delivery**	**Comparator**
Carter et al.	2009	Australia	Cohort	School	7–10 years, boys/girls with obesity/ overweight	17, 000 children over 4 years, (4 200 each year)	Yes: Parents involved but not costed	Multifaceted targeted programme, diet and physical activity	Treatment/ teacher	Usual care
Carter et al.	2009	Australia	RCT	Primary Care	10–11 years, boys/girls with obesity	5, 800 children	Yes: Parents involved but not costed	Primary care-based program, children with obesity and their parents, diet	Treatment/ GPs, paediatricians, psychologists, dietitians	Usual care
Hollingworth et al.	2012	UK	RCT	Hospital-community	4-5 years and 10-11 years, boys/girls with obesity/ overweight	9, 956 younger and 9, 698 older children	Yes: Parents involved but not costed	Interventions aimed at modifying behaviour, diet and/or physical activity	Treatment/GP, paediatricians, nurse, exercise specialist, dietitian	Usual care or minimal intervention
Moodie et al.	2008	Australia	RCT	Primary Care	5–9 years, boys/girls with obesity/ overweight	9, 685 children	Yes	Training of GP (3 times 2.5 h), 4 consultations over a 12-week period/physical activity, diet	Treatment/GP	Usual care

Notes: GP = general practitioner; RCT = randomised controlled trial

Tables S2-4 (i) – S2-4 (iv): Data extraction (Detailed account of the economic evaluation methods)

Table S2 (i) Detailed account of the economic evaluation methods A (trial-based prevention studies) (alphabetically sorted)

Authors and year	Measures of effectiveness/ study type	Type of modelling approach	Study perspective	Duration of intervention/ follow-up	Time horizon	Price year	Currency unit	Discount rate
Hayes et al. 2014	Reduction in BMI (z-score), unit BMI avoided/CEA	N.A.	Health care funder	2 years/ 2 years after randomisation	2 years	2012	AU$	Costs: 5% Effects: 5%
Kesztyus et al. 2011	Reduction in BMI, cm WC and unit WHtR prevented/CEA	N.A.	Societal	1 year/ 1 year after randomisation	1 year	2008	€	N.A.
Krauth et al. 2013	Reduction in BMI, (increase in physical activity: the measure was not specified)/CCA	N.A.	Societal	4 years/ 4, 5 and 6 years after intervention	6 years	No price year	€	Not stated
Martinez et al. 2011	Percent point decrease in triceps skinfold thickness and body fat/CEA	N.A.	Societal and institutional	8 months/ 8 months after randomisation	8 months	2005	€	N.A.
McAuley et al. 2010	Reduction in BMI (z-score), cm WC prevented, weight gain prevented, HRQoL/CEA	N.A.	Societal	2 years/ 2 and 4 years after intervention	4 years	2006	NZ$	Costs at 5% Effects: Not reported
Meng et al. 2013	Reduction in BMI, BMI (z-score), overweight and obesity case avoided/CEA	N.A.	Societal	1 year/ 1 year after randomisation	1 year	2010	RMB/ US$	N.A.
Peterson et al. 2008	(Increase in physical activity: the measure was not specified)/CEA	N.A.	Not specified	Not reported	Not reported	No price year	US$	N.A.
Sutherland et al. 2016	MVPA (min/day) gained, MET hours gained per person/day, BMI unit avoided and reduction in BMI (z-score)/CEA	N.A.	Societal	2 years/ 1 year (mid-intervention), 2 years after randomisation	2 years	2014	AU$	Not stated
Wang et al. 2008	Reduction in body fat/CEA	N.A.	Societal	1 year/ 1 year after randomisation	1 year	2003	US$	N.A.

Notes: BMI = body mass index; CCA = cost-consequence analysis; CEA = Cost-effectiveness analysis; HRQoL = health-related quality of life; MVPA = moderate-to-vigorous physical activity; WC = waist circumference; WHtR = waist-to-height ratio; N.A. = not applicable

Table S2 (ii) Detailed account of the economic evaluation methods A (trial-based treatment studies) (alphabetically sorted)

**Authors and year**	**Measures of effectiveness/ study type**	**Type of modelling approach**	**Study perspective**	**Duration of intervention/ follow-up**	**Time horizon**	**Price year**	**Currency unit**	**Discount rate**
Epstein et al. 2014	BMI change and weight for children and parents/CEA	N.A.	Societal (payer plus participant costs)	1 year/ 1 year after randomisation	1 year	No price year	US$	N.A.
Goldfield et al. 2001	Reduction in BMI (z-score) and percentage overweight/CEA	N.A.	Not specified	6 months/ 6 and 12 months after randomisation	1 year	No price year	$	N.A.
Hollinghurst et al. 2013	Reduction in BMI sd/CEA	N.A.	Healthcare	1 year/ 1 year after randomisation	1 year	No price year	£	N.A.
Janicke et al. 2009	Reduction in BMI/CEA	N.A.	Not specified	4 months/ 4 and 10 months after randomisation	10 months	No price year	US$	N.A.
Kalavainen et al. 2009	Reduction in weight for height and BMI/CEA	N.A.	Service provider (healthcare)	6 months/ 6 and 12 months after randomisation	1 year	2004	€	N.A.
Robertson et al. 2017	Reduction in waist z-score, body fat, WC, MVPA (min/day) gained, change in BMI (z-score) and QALYs gained/CEA, CUA	N.A.	NHS and PSS (healthcare)	3 months/ 3 and 12 months after randomisation	1 year	2013, 2014	£	N.A.
Wake et al. 2008	Reduction in BMI, parent-reported physical activity and dietary habits/CCA	N.A.	Societal	9 months/ 9 and 15 months after randomisation	15 months	2003	AU$	Not stated

Notes: BMI = body mass index; CCA = cost-consequence analysis; CEA = Cost-effectiveness analysis; CUA = cost-utility analysis; QALYs = quality-adjusted life years; MVPA = moderate-to-vigorous physical activity; NHS = National Health Service; PSS = personal social services; WC = waist circumference; N.A. = not applicable

Table S2 (iii) Detailed account of the economic evaluation methods A (model-based prevention studies) (alphabetically sorted)

Authors and year	Measures of effectiveness/ study type	Type of modelling approach	Study perspective	Duration of intervention/ follow-up	Time horizon	Price year	Currency unit	Discount rate
Barrett et al. 2015	Reduction in BMI and obesity-related healthcare expenditure, increase in minutes of MVPA and MET-hours/CEA	Markov model	Societal	2 years/ 2 years	10 years	2014	US$	Costs: 3% Effects: 3%
Brown et al. 2007	Cases of adult overweight prevented, QALYs saved/CUA	Decision analytic model	Societal	3 years/	25 years From age 40 to 64	2004	US$	Costs: 3% Effects: 3%
Carter et al. 2009 (4 the same)	BMI unit saved, DALYs saved/CUA	Markov model	Societal	1 year/	Lifetime	2001	AU$	Costs: 3% Effects: 3%
Graziose et al. 2016	Reduction in adult obesity, QALYs saved/CUA	Decision analytic model	Societal	1 year/	Lifetime	2012	US$	Costs: 3% Effects: 3%
Long et al. 2015	Changes in BMI, reductions in disease burden and healthcare expenditures, DALYs averted and QALYs gained/CEA, CUA	Markov model	Societal	2 years/ 2 years	10 years	2014	US$	Costs: 3% Effects: 3%
Magnus et al. 2009	BMI unit saved, DALYs saved/CEA, CUA	Markov model	Societal	1 year/	Lifetime	2001	AU$	Costs: 3% Effects: 3%
Moodie et al. 2009	BMI unit saved, DALYs saved, increase in physical activity (MET) and energy expenditure/CEA, CUA	Markov model	Societal	1 year/	Lifetime	2001	AU$	Costs: 3% Effects: 3%
Moodie et al. 2010	BMI unit saved, DALYs saved, increase in physical activity (MET) and energy expenditure/CEA, CUA	Markov model	Societal	1 year/	Lifetime	2001	AU$	Costs: 3% Effects: 3%

Table S2 (iii) Detailed account of the economic evaluation methods A (model-based prevention studies) (alphabetically sorted) continued

Moodie et al. 2011	BMI unit saved, DALYs saved, increase in physical activity (MET) and energy expenditure/CEA, CUA	Markov model	Societal	1 year/	Lifetime	2001	AU$	Costs: 3%<br>Effects: 3%
Moodie et al. 2013	Reduction in BMI, DALYs saved/CEA, CUA	Markov model	Societal	3 years/	Lifetime	2006	AU$	Costs: 3%<br>Effects: 3%
Pringle et al. 2010	Change in MPA, QALYs saved/CEA, CUA	Decision analytic model	Not specified	Not reported	Not reported	2003	£	N.A.
Rush et al. 2014	Reduction in BMI, QALYs saved, increased life expectancy/CUA	Markov model	Health treatment payer (Health care)	2 years/<br>5 years	Lifetime	2011	NZ$	Costs: 3.5%<br>Effects: 3.5%
Sonneville et al. 2015	Reduction in BMI, reductions in disease burden, healthcare expenditures and QALYs gained/CEA, CUA	Markov model	Societal	2 years/<br>2 years	10 years	2014	US$	Costs: 3%<br>Effects: 3%
Wang et al. 2003	Cases of adult overweight prevented, QALYs saved/CUA	Decision analytic model	Societal	2 years/	25 years From age 40 to 65	1996	US$	Costs: 3%<br>Effects: 3%
Wang et al. 2011	DWCB avoided, QALYs saved/CUA	Decision analytic model	Societal	2 years/	10 years	2010	US$	Costs: 3%<br>Effects: 3%
Wright et al. 2015	Unit BMI avoided, reduction in obesity-related healthcare expenditure/CEA	Markov model	Societal	2 years/<br>2 years	10 years	2014	US$	Costs: 3%<br>Effects: 3%

Notes: BMI = body mass index; CEA = Cost-effectiveness analysis; DALYs = disability-adjusted life years; DWCB = disordered weight control behaviours; QALYs = quality-adjusted life years; MVPA = moderate-to-vigorous physical activity; MPA = moderate physical activity; N.A. = not applicable

Table S2 (iv) Detailed account of the economic evaluation methods A (model-based treatment studies) (alphabetically sorted)

Authors and year	Measures of effectiveness/ study type	Type of modelling approach	Study perspective	Duration of intervention/ follow-up	Time horizon	Price year	Currency unit	Discount rate
Carter et al. 2009 (2 the same)	BMI unit saved, DALYs saved/CUA	Markov model	Societal	1 year/	Lifetime	2001	AU$	Costs: 3% Effects: 3%
Hollingworth et al. 2012	Reduction in BMI sd, life year gained/CEA	Markov model	NHS (healthcare)	1 year/	Lifetime	2009	£	Costs: 3% Effects: 3%
Moodie et al. 2008	BMI unit saved, DALYs saved/CEA, CUA	Markov model	Societal	1 year/	Lifetime	2001	AU$	Costs: 3% Effects: 3%

Notes: BMI = body mass index; CEA = Cost-effectiveness analysis; CUA = cost-utility analysis; DALYs = disability-adjusted life years; NHS = National Health Service; NHF = National Health Forum

Table S3 (i) Detailed account of the economic evaluation methods B (trial-based prevention studies) (alphabetically sorted)

Authors and year	Methods for estimating/collecting resource use	Cost categories	Largest cost drivers	Excluded costs	Average costs per participant	Funding source
Hayes et al. 2014	Local health district records, patient-level data linkage	Programme delivery, direct medical	Hospitalisation and doctor visits	Research and development, birth, evaluation or administration of the clinical trial	AU$ 1, 309	Academic
Kesztyus et al. 2011	Official statistics of the state of Bavaria	Programme delivery	Scientific coordinator	Development, scientific evaluation, classroom time	€ 24.09	Academic
Krauth et al. 2013	Questionnaire, school admin	Programme delivery, indirect	Training	Not stated	€ 619	Academic
Martinez et al. 2011	Not stated	Programme delivery, labour	Personnel (coordinator)	Parents' care costs	€ 269.83	Academic
McAuley et al. 2010	Not stated	Programme delivery	coordinator	Research and development, planning phase, time costs of the children and their parents	NZ$ 1, 281	Academic
Meng et al. 2013	Not stated	Programme delivery, labour, money, evaluation	Materials	Not stated	Combined: RMB 182.4 (US$ 26.8), nutrition: RMB 52.8 (US$ 7.8), PA: RMB 52.3 (US$ 7.7)	Academic

Table S3 (i) Detailed account of the economic evaluation methods B (trial-based prevention studies) (alphabetically sorted) continued

Peterson et al. 2008	Not stated	Development, media production and placement	Not stated	Not stated	Per person to become more active: Individual sections: US$ 5.11- 153.19 Whole: US$ 8.87	Not stated
Sutherland et al. 2016	Using market rates, Australian Bureau of Statistics, Industrial Relations Commission of NSW/project records	Programme delivery	Consultant	Research and development, potential effects on healthcare costs	AU$ 394	Academic
Wang et al. 2008	Not stated	Programme delivery, usual after-school care costs without intervention, indirect	Personnel	Not stated	US$ 956	Academic

Table S3 (ii) Detailed account of the economic evaluation methods B (trial-based treatment studies) (alphabetically sorted)

**Authors and year**	**Methods for estimating/collecting resource use**	**Cost categories**	**Largest cost drivers**	**Excluded costs**	**Average costs per participant**	**Funding source**
Epstein et al. 2014	Tracking and recording by staff members, Google maps calculations	Programme delivery, direct medical, direct non-medical, indirect	Treatment time	Recruitment	Cost per family: FBT: US$ 1, 448, PC-1: US$ 2, 260, PC-2: US$ 2, 124	Academic
Goldfield et al. 2001	Not stated	Programme delivery, direct medical	Salary	Reduced cost of medical care, purchasing new clothes, time costs for being physically active	Cost per family: group treatment: US$ 491 Mixed treatment: US$ 1, 390	Academic
Hollinghurst et al. 2013	Patient-level data linkage	Programme delivery, direct medical	Mandometer device	Development of the Mandometer and staff training	Mandometer group: £ 1, 749 (SD £ 243), primary care group: £ 301 (SD £76), hospital groups: £ 263 (SD £ 88) and £ 209 (SD £ 81)	Academic
Janicke et al. 2009	Not stated	Programme delivery, direct medical	Group leaders	Research (assessment, recruitment), participants (travel, purchasing healthier foods)	Family-based group: US$ 872, Parent-only: US$ 521	Academic

Table S3 (ii) Detailed account of the economic evaluation methods B (trial-based treatment studies) (alphabetically sorted) continued

Kalavainen et al. 2009	Not stated	Programme delivery, labour, direct medical	Labour	Research component, participating families	Cost per family: group treatment: € 327, routine counselling: € 61	Academic
Robertson et al. 2017	Questionnaire and secondary national tariff sets	Programme delivery, direct medical, indirect	Hospital visits, salary (GP)	Not stated	£ 998	Academic
Wake et al. 2008	3 main sources: the LEAP team records, practice audit, and parent written questionnaires at 9 months	Programme delivery, direct medical, direct non-medical, indirect	Practice	Set-up, research and development, training	AU$ 705	Academic

Table S3 (iii) Detailed account of the economic evaluation methods B (model-based prevention studies) (alphabetically sorted)

Authors and year	Methods for estimating/collecting resource use	Cost categories	Largest cost drivers	Excluded costs	Average costs per participant	Funding source
Barrett et al. 2015	Beta, normal or uniform distribution form, different databases (school administrators, interventions, survey)	Programme delivery, avoided direct medical	Sets (nationally) of active PE curricula and equipment	Start-up	US$ 4.03	Academic
Brown et al. 2007	Not stated	Programme delivery, avoided direct medical, avoided indirect (productivity loss)	Promotional	Not stated	US$ 104	Academic
Carter et al. 2009	Not stated	Programme delivery	Not stated	Set-up	AU$ 28	Academic
Carter et al. 2009	Not stated	Programme delivery	Not stated	Set-up, Teacher classroom time	AU$ 473	Academic
Carter et al. 2009	Not stated	Programme delivery	Not stated	Set-up, Teacher classroom time	AU$ 103	Academic
Carter et al. 2009	Not stated	Programme delivery	Not stated	Set-up, Teacher classroom time	AU$ 211	Academic
Graziose et al. 2016	New York City Department of Education (NYCDOE) and author estimate	Programme delivery, future obesity-related medical, avoided direct medical	Teacher preparation time	Development and evaluation	US$ 111	Academic

Table S3 (iii) Detailed account of the economic evaluation methods B (model-based prevention studies) (alphabetically sorted) continued

Long et al. 2015	Beta, normal or uniform distribution form, different databases (interventions, revenue department, bureau of labour statistics 2013)	Programme delivery, labour, avoided direct medical	Industry auditor salary	Not stated	US$ 0.68	Academic
Magnus et al. 2009	Not stated	Programme delivery, other sectors	Government regulators	Set-up	AU$ 0.54	Academic
Moodie et al. 2009	Middle of Australian public service Level 6, Australian bureau of statistics and Victorian department of education and training	Programme delivery, direct non-medical, indirect, other sectors,	Education e.g. programme coordinator	Set-up, research and development implementation	AU$ 2, 908	Academic
Moodie et al. 2010	Middle of Australian public service level 6 and Victorian department of education and training	Programme delivery, indirect, other sectors	Sport and recreation	Set-up, research and development, implementation, external evaluation and maintenance	AU$ 488.5	Academic
Moodie et al. 2011	Middle of Australian public service level 6, Victorian department of education and training	Programme delivery, other sectors	Education e.g. Central coordinator	Set-up, research and development	AU$ 49.68	Academic
Moodie et al. 2013	Australian bureau of statistics, Victorian department of education and training, etc.	Programme delivery, direct non-medical, indirect, other sectors	Personnel time	Student time, spin-off activities, changes in the physical activity and eating patterns of participating families	AU$ 344	Academic
Pringle et al. 2010	Not stated	Programme delivery,	Primary care referral	Not stated	_	Academic

Table S3 (iii) Detailed account of the economic evaluation methods B (model-based prevention studies) (alphabetically sorted) continued

Rush et al. 2014	Not stated	Programme delivery, avoided direct medical	Not stated	Set-up and development, indirect, out-of-pocket,	NZ$ 44.96	Academic
Sonneville et al. 2015	Normal or beta distribution form, different databases (bureau of labour statistics 2013, etc)	Programme delivery, labour, avoided direct medical	Industry auditor salary	Not stated	US$ 0.015	Academic
Wang et al. 2003	Not stated	Programme delivery, avoided direct medical, avoided indirect (productivity loss)	Subject teachers	Classroom time	US$ 28	Academic
Wang et al. 2011	Not stated	Programme delivery, avoided direct medical	Subject teachers	Not stated	US$ 184.27	Academic
Wright et al. 2015	Different databases (bureau of labour statistics 2013, etc)	Programme delivery, avoided direct medical	Supervising and training	Not stated	US$ 1.29	Academic

Table S3 (iv) Detailed account of the economic evaluation methods B (model-based treatment studies) (alphabetically sorted)

Authors and year	Methods for estimating/collecting resource use	Cost categories	Largest cost drivers	Excluded costs	Average costs per participant	Funding source
Carter et al. 2009	Not stated	Programme delivery	Not stated	Set-up	AU$ 129	Academic
Carter et al. 2009	Not stated	Programme delivery	Not stated	Set-up,	AU$ 1,896	Academic
Hollingworth et al. 2012	Not stated	Programme delivery, lifetime treatment, obesity-related diseases	Salary (GP)	Not stated	£108 - 662	Academic
Moodie et al. 2008	Middle of Australian public service Level 6, LEAP trial, etc.	Programme delivery, direct medical, direct non-medical, indirect	Project coordinator	Set-up, research and development, resultant changes in patient behaviour	AU$ 650.5	Academic

Table S4 (i) Detailed account of the economic evaluation methods C (trial-based prevention studies) (alphabetically sorted)

Authors and year	ICER/average cost per benefit	Uncertainty analysis	Sensitivity analysis type	Sensitivity analysis	Cost-effective
Hayes et al. 2014	AU$ 4, 230 per unit BMI avoided,<br>AU$ 631 per 0.1 reduction in BMI (z-score)	_	DSA	Adjustments in nurse travel time	Likely to be
Kesztyus et al. 2011	€ 11.11 per WC cm prevented<br>€ 18.55 per WHtR unit prevented	_	DSA	Teachers individual working time to prepare the lessons, difference in effects tested at a 10, 20 and 30% lower value	Likely to be
Krauth et al. 2013	N.A.	_	DSA	_	N.A.
Martinez et al. 2011	ICER: No<br>€ 500 per 1% decrease in triceps skinfold thickness	_	DSA	Differences in costs (modification of the venue cost)	Likely to be
McAuley et al. 2010	ICER: No<br>NZ$ 664–1708 per kg of weight gain prevented (depending on age),	_	DSA	Differences in weight z-score (ranged from 0.5 to 1.0 in the youngest children and 1.4 to 2.4 in the oldest children)	Likely to be
Meng et al. 2013	Combined intervention:<br>US$ 120.3 per 1 kg/m2 BMI reduction,<br>US$ 249.3 per BMI z-score (BAZ),<br>US$ 1308.9 per one overweight and obesity case avoided	_	Not stated	N.A.	Likely to be
Peterson et al. 2008	ICER: No<br>Entire campaign:<br>US$ 4.01: to see the ad,<br>US$ 7.35: to consider being more active,<br>US$ 8.87: actually become more active,<br>with bill-boards the most cost-effectiveness	_	Not stated	N.A.	Likely to be

Table S4 (i) Detailed account of the economic evaluation methods C (trial-based prevention studies) (alphabetically sorted) continued

Sutherland et al. 2016	AU$ 56 per additional minute of MVPA,<br>AU$ 1 per MET hour gained per person per day,<br>AU$ 1, 408 per BMI unit avoided,<br>AU$ 563 per 10 % reduction in BMI z-score	_	DSA	Higher and lower estimate of the assumed opportunity cost, varying the magnitude of the effect size, extending the benefit of physical activity recess and lunchtime activities to students beyond the target year, extending the benefit of multiple strategies to all students<br>Scenario: State wide rollout (current model), state wide roll out – Alternative (real world) model	Likely to be
Wang et al. 2008	US$ 317 per 0.76% body fat reduction	_	DSA	Changing the per capita usual after-school care costs (ranging from US$ 5.00 to US$ 10.00)	Likely to be

Notes: BAZ = BMI (z-score); BMI = body mass index; DSA = deterministic sensitivity analysis; MVPA = moderate-to-vigorous physical activity; WC = waist circumference; WHtR = waist-to-height ratio; N.A. = not applicabl

Table S4 (ii) Detailed account of the economic evaluation methods C (trial-based treatment studies) (alphabetically sorted)

Authors and year	ICER/average cost per benefit	Uncertainty analysis	Sensitivity analysis type	Sensitivity analysis	Cost-effective
Epstein et al. 2014	ICER: No<br>Children:<br>FBT US$ 209.17 per % over BMI,<br>PC1 US$ 1, 036.50 per % over BMI,<br>PC2 US$ 973.98 per % over BMI,<br>Parents:<br>FBT US$ 132.97 per pound (lb),<br>PC1 US$ 373.53 per pound (lb),<br>PC2 US$ 351.00 per pound (lb)	_	Not stated	N.A.	Likely to be
Goldfield et al. 2001	ICER: No<br>US$ 1, 000 per 10% overweight reduction<br>US$ 1, 000 per 0.6 decrease in BMI z-score	_	Not stated	N.A.	Likely to be
Hollinghurst et al. 2013	£ 432 per 0.1 reduction in BMI sd	_	Not stated	N.A.	Likely to be
Janicke et al. 2009	ICER: No<br>Family-based group:<br>US$ 758 per 0.10 decrease in BMI z-score,<br>Parent-only:<br>US$ 579 per 0.10 decrease in BMI z-score	_	Not stated	N.A.	Likely to be
Kalavainen et al. 2009	€ 53 per 1% decrease in weight for height<br>€ 266 per 0.1 decrease in BMI	_	DSA	Group treatment costs: salaries of two group leaders included in costs	Likely to be

Table S4 (ii) Detailed account of the economic evaluation methods C (trial-based treatment studies) (alphabetically sorted) continued

Robertson et al. 2017	£ 552, 175 per QALY saved,<br>£ - 3, 935 per unit change in BMI (z-score)	_	DSA	'programme completers': families that participated in 5 or more sessions, multiple imputation of all missing cost and outcomes data, alternative sources and inputs for EQ-5D utility values	Unlikely to be
Wake et al. 2008	N.A.	_	DSA	Baseline: Value of parents' time, equal parent's time, unit cost of GP visit, economies of scale<br>Combinations:	N.A.

Notes: BMI = body mass index; DSA = deterministic sensitivity analysis; FBT = family-based behavioural treatment; QALYs = quality-adjusted life years; PC = parent and child; N.A. = not applicabl

Table S4 (iii) Detailed account of the economic evaluation methods C (model-based prevention studies) (alphabetically sorted)

Authors and year	ICER/average cost per benefit	Uncertainty analysis	Sensitivity analysis type	Sensitivity analysis	Cost-effective
Barrett et al. 2015	US$ 401 per unit BMI avoided: 2 years<br>US$ 1, 720 per BMI unit reduced: 10 years<br>Reduction of healthcare costs by $ 60.5 million: 10 years	PSA	DSA, PSA	Physical activity and BMI changes, more PE time, cost of intervention	Likely to be
Brown et al. 2007	US$ 900 per QALY saved	_	PSA	Both overall and Hispanics (Cases of adult overweight prevented, QALYs saved, medical costs averted, costs of lost labour productivity averted	Likely to be
Carter et al. 2009	AU$ 5, 000 per DALY saved	PSA	DSA, PSA	_	Dominant
Carter et al. 2009	AU$ 1, 800 per DALY saved	PSA	DSA, PSA	_	Dominant
Carter et al. 2009	AU$ 5, 100 per DALY saved	PSA	DSA, PSA	_	Dominant
Carter et al. 2009	AU$ 5, 600 per DALY saved	PSA	DSA, PSA	_	Likely to be
Graziose et al. 2016	US$ 275 per QALY saved	PSA	DSA, PSA	Relapse into adulthood, intervention is effective only for Hispanic and black students, intervention is effective only for male students	Likely to be

Table S4 (iii) Detailed account of the economic evaluation methods C (model-based prevention studies) (alphabetically sorted) continued

Long et al. 2015	US$ 8.54 per unit BMI avoided: 2 years,	PSA	DSA, PSA	Change in SSB consumption and BMI, cost of implementing SSB excise tax	Dominant
Magnus et al. 2009	AU$ 5.00 per BMI unit saved, AU$ 3.70 per DALY saved	PSA	DSA, PSA	BMI and cost changes	Dominant
Moodie et al. 2009	AU$ 87, 000 per BMI unit saved, AU$ 760, 000 per DALY saved	PSA	DSA, PSA	Reduce costs, improve capacity utilisation and recruitment, increase participants receiving benefit, combine scenarios	Unlikely to be
Moodie et al. 2010	AU$ 8, 200 per BMI unit saved, AU$ 82, 000 per DALY saved	PSA	DSA, PSA	Reduction in the number of sites and co-ordinators, application of the same wage rate to all site co-ordinators (school, OSHC), combination scenarios, all participants receive full intervention benefit	Unlikely to be
Moodie et al. 2011	AU$ 13, 000 per BMI unit saved, AU$ 117, 000 per DALY saved	PSA	DSA, PSA	joint cost attribution across multiple objectives, broadening of the benefit to include other children in the school, exclusion of selected costs items	Unlikely to be
Moodie et al. 2013	AU$ 576 per BMI unit saved, AU$ 29, 798 per DALY saved	PSA	DSA, PSA	Alternative decay of effect, if only 50% of children received the benefit	Likely to be
Pringle et al. 2010	ICER: No<br>£ 47 - 509 per QALY gained,<br>£ 260 - 2, 786 per completer improving at least one MPA	_	Not stated	N.A.	Dominant

Table S4 (iii) Detailed account of the economic evaluation methods C (model-based prevention studies) (alphabetically sorted) continued

Rush et al. 2014	NZ$ 24, 690 per QALY saved: older children NZ$ 30, 438 per QALY saved: younger children	_	DSA, PSA	Varied conditions for younger and older children (varying of the cost of intervention, BMI, the annual discount rate and the horizon of the model)	Likely to be
Sonneville et al. 2015	US$ 1.16 per unit BMI avoided: 2 years,	PSA	DSA, PSA	Differences in BMI associated with the number of fast food advertising messages seen, cost of intervention	Dominant
Wang et al. 2003	US$ 4, 035 per QALY saved	_	DSA, PSA	Cases of adult overweight prevented, years of healthy life, annual discount rate, medical care costs averted, annual workdays lost averted	Dominant
Wang et al. 2011	US$ 2, 966 per QALY saved	PSA	DSA, PSA	Percentage of girls with DWCB who had SED, progression probability, long-term medical costs per BN patients, HRQoL of BN patients, time to recovery	Dominant
Wright et al. 2015	US$ 57.80 per BMI unit avoided: 2 years Net healthcare cost savings of $ 51.6 million: 10 years	PSA	DSA, PSA	Time spent in care, alternative policy adherence estimates and outcomes	Dominant

Notes: BMI = body mass index; BN = Bulimia Nervosa; DALYs = disability-adjusted life years; DSA = deterministic sensitivity analysis; DWCB = disordered weight control behaviours; HRQoL = health-related quality of life; QALYs = quality-adjusted life years; MPA = moderate physical activity; PE = physical education; PSA = probabilistic sensitivity analysis; SED = sub-diagnostic eating disorders; SSB = sugar sweetened beverage; N.A. = not applicable

Table S4 (iv) Detailed account of the economic evaluation methods C (model-based treatment studies) (alphabetically sorted)

Authors and year	ICER/average cost per benefit	Uncertainty analysis	Sensitivity analysis type	Sensitivity analysis	Cost-effective
Carter et al. 2009	AU$ 3, 300 per DALY saved	PSA	DSA, PSA	_	Dominant
Carter et al. 2009	AU$ 1, 500 per DALY saved	PSA	DSA, PSA	_	Dominant
Hollingworth et al. 2012	£ 400 per 0.13 reduction in BMI sd, £ 13, 589 per life year gained	_	DSA	BMI sd (minimal, median, or maximal effect size) and intervention cost (low, moderate and high)	Dominant
Moodie et al. 2008	AU$ 4, 670 per DALY saved	PSA	DSA, PSA	Full maintenance of the BMI benefit into adulthood/vs. half maintenance, outlier removal, delivery of intervention, (family attendance, etc), recruitment rates	Likely to be

Notes: BMI = body mass index; DALYs = disability-adjusted life years; DSA = deterministic sensitivity analysis; PSA = probabilistic sensitivity analysis

Table S5: Drummond checklist for critically appraising relevant studies

Table S5 Drummond checklist

**Drummond checklist for assessing primary economic evaluations**				
**Study design**				
1 The research question is stated				
2 The economic importance of the research question is stated				
3 The viewpoint (s) of the analysis are clearly stated and justified				
4 The rationale for choosing alternative programmes or interventions compared is stated				
5 The alternatives being compared are clearly described				
6 The form of economic evaluation used is stated				
7 The choice of form of economic evaluation is justified in relation to the questions addressed				
**Data collection**				
8 The source (s) of effectiveness estimates used are stated				
9 Details of the design and results of effectiveness study are given (if based on a single study)				
10 Details of the methods of synthesis or meta-analysis of estimates are given (if based on a synthesis of a number of effectiveness studies)				
11 The primary outcome measure (s) for the economic evaluation are clearly stated				
12 Methods to value benefits are stated				
13 Details of the subjects from whom valuations were obtained were given				
14 Productivity changes (if included) are reported separately				
15 The relevance of productivity changes to the study question is discussed				
16 Quantities of resource use are reported separately from their unit costs				
17 Methods for the estimation of quantities and unit costs are described				
18 Currency and price data are recorded				
19 Details of currency of price adjustments for inflation or currency conversion are given				
20 Details of any model used are given				
21 The choice of model used and the key parameters on which it is based are justified				
**Analysis and interpretation of results**				

22 Time horizon of costs and benefits is stated				
23 The discount rate (s) is stated				
24 The choice of discount rate (s) is justified				
25 An explanation is given if costs and benefits are not discounted				
26 Details of statistical tests and confidence intervals are given for stochastic data				
27 The approach to sensitivity analysis is given				
28 The choice of variables for sensitivity analysis is justified				
29 The ranges over which the variables are varied are justified				
30 Relevant alternatives are compared				
31 Incremental analysis is reported				
32 Major outcomes are presented in a disaggregated as well as aggregated form				
33 The answer to the study question is given				
34 Conclusions follow from the data reported				
35 Conclusions are accompanied by the appropriate caveats				

Notes: Y= Yes; N=No; NC= Not clear; N.A. = Not applicable

Tables S6 (i) – S6 (iv): Quality assessment of the included studies

Table S6 (i) Critically appraising trial-based prevention studies (alphabetically sorted)

Drummond Checklist	Hayes et al. 2014	Kesztyus et al. 2011	Krauth et al. 2013	Martinez et al. 2011	McAuley et al. 2010	Meng et al. 2013	Peterson et al. 2008	Sutherland et al. 2016	Wang et al. 2008
**Study design**									
1	Y	Y	Y	Y	Y	Y	Y	Y	Y
2	Y	Y	Y	Y	NC	Y	NC	Y	NC
3	Y	Y	Y	Y	Y	Y	Y	Y	Y
4	Y	NC	N	NC	N	N	N	NC	N
5	Y	Y	Y	Y	Y	Y	NC	Y	Y
6	Y	Y	Y	Y	Y	Y	Y	Y	Y
7	Y	N	N	N	Y	NC	Y	Y	NC
**Data collection**									
8	Y	Y	Y	Y	Y	Y	Y	Y	Y
9	Y	Y	Y	Y	Y	Y	NC	Y	Y
10	N.A.	N.A.	N.A.	N.A.	N.A.	N.A.	N.A.	N.A.	N.A.
11	Y	Y	Y	Y	Y	Y	NC	Y	Y
12	N.A.	Y	Y	Y	Y	Y	N.A.	Y	Y
13	N.A.	Y	Y	Y	N	Y	N.A.	Y	N
14	N.A.	NC	N	NC	N	N	N.A.	NC	N
15	N.A.	N	N	N	N	N	N.A.	N	N
16	Y	Y	NC	Y	Y	N	N	Y	Y
17	Y	Y	NC	Y	NC	N	N	Y	NC
18	Y	Y	NC	Y	Y	Y	NC	Y	Y
19	Y	N	N.A.	N	Y	N	N	N	N
20	N.A.	N.A.	N.A.	N.A.	N.A.	N.A.	N.A.	N.A.	N.A.
21	N.A.	N.A.	N.A.	N.A.	N.A.	N.A.	N.A.	N.A.	N.A.
**Analysis and interpretation of results**									
22	Y	Y	Y	Y	Y	Y	N	Y	Y
23	Y	N.A.	N.A.	N.A.	NC	N.A.	N.A.	N	N.A.
24	Y	N.A.	N.A.	N.A.	NC	N.A.	N.A.	N.A.	N.A.
25	N.A.	N.A.	N.A.	N.A.	N	N.A.	N.A.	N	N.A.

26	Y	Y	N	NC	Y	Y	N	Y	NC
27	Y	Y	Y	Y	Y	N	N	Y	Y
28	Y	N	N	N	NC	N.A.	N.A.	Y	NC
29	Y	N	N	N	NC	N.A.	N.A.	Y	NC
30	Y	Y	N	Y	Y	N.A.	N.A.	Y	Y
31	Y	Y	N.A.	N	N	Y	N	Y	Y
32	Y	Y	N.A.	N	N	Y	N	Y	Y
33	Y	Y	Y	Y	Y	Y	Y	Y	Y
34	Y	Y	Y	Y	Y	Y	Y	Y	Y
35	Y	Y	Y	Y	Y	Y	NC	Y	Y

Notes: Y= Yes; N=No; NC= Not clear; N.A. = Not applicable

Table S6 (ii) Critically appraising trial-based treatment studies (alphabetically sorted)

Drummond Checklist	Epstein et al. 2014	Goldfield et al. 2001	Hollinghurst et al. 2013	Janicke et al. 2009	Kalavainen et al. 2009	Robertson et al. 2017	Wake et al. 2008
**Study design**							
1	Y	Y	Y	P	Y	Y	Y
2	Y	NC	Y	Y	Y	Y	Y
3	Y	Y	Y	Y	Y	Y	Y
4	Y	N	N	N	N	NC	N
5	Y	Y	Y	Y	Y	Y	NC
6	Y	Y	Y	Y	Y	Y	Y
7	NC	N	NC	N	Y	NC	Y
**Data collection**							
8	Y	Y	Y	Y	Y	Y	Y
9	Y	Y	Y	Y	Y	Y	Y
10	N.A.	N.A.	N.A.	N.A.	N.A.	N.A.	N.A.
11	Y	Y	Y	Y	Y	Y	Y
12	Y	N.A.	N.A.	N.A.	N.A.	N.A.	Y
13	Y	N.A.	N.A.	N.A.	N.A.	N.A.	NC
14	NC	N.A.	N.A.	N.A.	N.A.	N.A.	NC
15	N	N.A.	N.A.	N.A.	N.A.	N.A.	N
16	N	NC	Y	Y	Y	NC	NC
17	N	N	Y	N	N	Y	NC
18	NC	NC	NC	NC	Y	Y	Y
19	N	N	N	N	N	Y	N.A.
20	N.A.	N.A.	N.A.	N.A.	N.A.	N.A.	N.A.
21	N.A.	N.A.	N.A.	N.A.	N.A.	N.A.	N.A.
**Analysis and interpretation of results**							
22	Y	Y	Y	Y	Y	Y	Y
23	N.A.	N.A.	N.A.	N.A.	N.A.	N.A.	N.A.
24	N.A.	N.A.	N.A.	N.A.	N.A.	N.A.	N.A.
25	N.A.	N.A.	N.A.	N.A.	N.A.	N.A.	N.A.

26	Y	Y	NC	NC	Y	Y	NC
27	N	N	N	N	Y	Y	Y
28	N.A.	N.A.	N.A.	N.A.	NC	Y	Y
29	N.A.	N.A.	N.A.	N.A.	NC	Y	Y
30	N.A.	N.A.	N.A.	N.A.	Y	Y	Y
31	N	N	Y	N	Y	Y	N.A.
32	N	N	Y	N	Y	Y	N.A.
33	Y	Y	Y	Y	Y	Y	Y
34	Y	Y	Y	Y	Y	Y	Y
35	Y	Y	Y	Y	Y	Y	Y

Notes: Y= Yes; N=No; NC= Not clear; N.A. = Not applicable

Table S6 (iii) Critically appraising model-based prevention studies (alphabetically sorted)

Drummond Checklist	Barrett et al. 2015	Brown et al. 2007	Carter et al. 2009	Graziose et al. 2016	Long et al. 2015	Magnus et al. 2009	Moodie et al. 2009	Moodie et al. 2010
**Study design**								
1	Y	Y	Y	Y	Y	Y	Y	Y
2	Y	NC	NC	Y	Y	Y	Y	N
3	Y	Y	Y	Y	Y	Y	Y	Y
4	N	N	N	Y	N	N	N	Y
5	Y	Y	Y	Y	Y	Y	Y	Y
6	Y	Y	Y	Y	Y	Y	Y	Y
7	NC	N	Y	Y	NC	N	N	N
**Data collection**								
8	Y	Y	Y	Y	Y	Y	Y	Y
9	Y	Y	Y	Y	Y	Y	Y	Y
10	N.A.	N.A.	N.A.	N.A.	N.A.	N.A.	N.A.	N.A.
11	Y	Y	Y	Y	Y	Y	Y	Y
12	Y	Y	Y	Y	Y	Y	Y	Y
13	NC	NC	NC	NC	NC	N	NC	NC
14	N	NC	N	NC	N	N	NC	NC
15	N	N	N	N	N	N	N	N
16	Y	NC	N	Y	Y	N	Y	NC
17	Y	NC	N	Y	Y	N	Y	Y
18	Y	Y	Y	Y	Y	Y	Y	Y
19	N	N	Y	N	N	N	Y	Y
20	Y	NC	NC	Y	Y	NC	NC	NC
21	NC	N	N	N	NC	N	N	N
**Analysis and interpretation of results**								
22	Y	Y	Y	Y	Y	Y	Y	Y
23	Y	Y	Y	Y	Y	Y	Y	Y
24	N	N	Y	Y	N	N	Y	Y
25	N.A.	N.A.	N.A.	N.A.	N.A.	N.A.	N.A.	N.A.

26	Y	NC	Y	Y	Y	NC	Y	Y
27	Y	Y	Y	Y	Y	Y	Y	Y
28	Y	Y	N	N	N	N	Y	Y
29	Y	Y	N	N	N	N	Y	Y
30	Y	Y	N	Y	Y	Y	Y	Y
31	Y	Y	Y	Y	Y	Y	Y	Y
32	Y	Y	Y	Y	Y	Y	Y	Y
33	Y	NC	Y	Y	Y	Y	Y	Y
34	Y	Y	Y	Y	Y	Y	Y	Y
35	Y	NC	NC	Y	Y	Y	Y	Y

Notes: Y= Yes; N=No; NC= Not clear; N.A. = Not applicable

Table S6 (iii) Critically appraising model-based prevention studies (alphabetically sorted) continued

Drummond Checklist	Moodie et al. 2011	Moodie et al. 2013	Pringle et al. 2010	Rush et al. 2014	Sonneville et al. 2015	Wang et al. 2003	Wang et al. 2011	Wright et al. 2015
**Study design**								
1	Y	Y	Y	Y	Y	Y	Y	Y
2	N	Y	NC	Y	Y	NC	Y	Y
3	Y	Y	Y	Y	Y	Y	Y	Y
4	Y	Y	N	NC	N	N	N	N
5	Y	Y	NC	Y	Y	Y	Y	Y
6	Y	Y	Y	Y	Y	Y	Y	Y
7	Y	Y	NC	Y	NC	NC	NC	NC
**Data collection**								
8	Y	Y	Y	Y	Y	Y	Y	Y
9	Y	Y	NC	Y	Y	Y	NC	Y
10	N.A.	N.A.	N.A.	N.A.	N.A.	N.A.	N.A.	N.A.
11	Y	Y	NC	Y	Y	Y	Y	Y
12	Y	Y	N.A.	N.A.	Y	Y	Y	Y
13	NC	Y	N.A.	N.A.	NC	NC	NC	NC
14	NC	Y	N.A.	N.A.	NC	N	NC	N
15	N	Y	N.A.	N.A.	N	N	N	N
16	Y	Y	NC	N	Y	Y	Y	Y
17	Y	Y	NC	NC	Y	NC	NC	Y
18	Y	Y	Y	Y	Y	Y	Y	Y
19	Y	Y	N	N	N	N	N	N
20	NC	NC	NC	NC	Y	Y	NC	Y
21	N	N	N	N	NC	N	N	NC
**Analysis and interpretation of results**								
22	Y	Y	N	Y	Y	Y	Y	Y
23	Y	Y	N	Y	Y	Y	Y	Y
24	Y	Y	N.A.	N	N	N	N	N
25	N.A.	N.A.	N	N.A.	N.A.	N.A.	N.A.	N.A.

26	Y	Y	N	Y	Y	Y	Y	Y
27	Y	Y	Y	Y	Y	Y	Y	Y
28	Y	NC	N	NC	N	Y	NC	Y
29	Y	NC	N	NC	N	Y	NC	Y
30	Y	Y	N.A.	Y	Y	Y	Y	Y
31	Y	Y	N	Y	Y	Y	Y	Y
32	Y	Y	N	Y	Y	Y	Y	Y
33	Y	Y	NC	Y	Y	NC	Y	Y
34	Y	Y	Y	Y	Y	Y	Y	Y
35	Y	Y	NC	Y	Y	Y	Y	Y

Notes: Y= Yes; N=No; NC= Not clear; N.A. = Not applicable

Table S6 (iv) Critically appraising model-based treatment studies (alphabetically sorted)

Drummond Checklist	Carter et al. 2009	Hollingworth et al. 2012	Moodie et al. 2008
**Study design**			
1	Y	Y	Y
2	NC	Y	Y
3	Y	Y	Y
4	N	N	Y
5	Y	Y	Y
6	Y	Y	Y
7	Y	NC	Y
**Data collection**			
8	Y	Y	Y
9	Y	Y	Y
10	N.A.	N.A.	N.A.
11	Y	Y	Y
12	Y	N.A.	Y
13	NC	N.A.	Y
14	N	N.A.	Y
15	N	N.A.	N
16	N	N	Y
17	N	N	Y
18	Y	Y	Y
19	Y	N	Y
20	NC	NC	NC
21	N	NC	N
**Analysis and interpretation of results**			
22	Y	Y	Y
23	Y	Y	Y
24	Y	N	Y
25	N.A.	N.A.	N.A.

26	Y	NC	Y
27	Y	Y	Y
28	N	N	Y
29	N	N	Y
30	N	Y	Y
31	Y	Y	Y
32	Y	Y	Y
33	Y	Y	Y
34	Y	Y	Y
35	NC	Y	Y

Notes: Y= Yes; N=No; NC= Not clear; N.A. = Not applicable
